# Unraveling volatile metabolites in pigmented onion (*Allium cepa* L.) bulbs through HS-SPME/GC–MS-based metabolomics and machine learning

**DOI:** 10.3389/fnut.2025.1582576

**Published:** 2025-04-22

**Authors:** Kaiqi Cheng, Jingzhe Xiao, Jingyuan He, Rongguang Yang, Jinjin Pei, Wengang Jin, A. M. Abd El-Aty

**Affiliations:** ^1^Qinba State Key Laboratory of Biological Resource and Ecological Environment (Incubation), Collaborative Innovation Center of Bio-Resource in Qinba Mountain Area, Shaanxi University of Technology, Hanzhong, China; ^2^Key Laboratory of Bio-Resources of Shaanxi Province, School of Bioscience and Engineering, Shaanxi University of Technology, Hanzhong, China; ^3^Department of Pharmacology, Faculty of Veterinary Medicine, Cairo University, Giza, Egypt; ^4^Department of Medical Pharmacology, Medical Faculty, Ataturk University, Erzurum, Türkiye

**Keywords:** pigmented onion, volatile flavor, metabolomics, machine learning models, HS-SPME/GC–MS

## Abstract

**Introduction:**

Colored onions are favored by consumers due to their distinctive aroma, rich phytochemical content, and diverse biological activities. However, comprehensive analyses of their phytochemical profiles and volatile metabolites remain limited.

**Methods:**

In this study, total phenols, flavonoids, anthocyanins, carotenoids, and antioxidant activities of three colored onion bulbs were evaluated. Volatile metabolites were identified using headspace solid-phase microextraction combined with gas chromatography-mass spectrometry (HS-SPME/GC-MS). Multivariate statistical analyses, feature selection techniques (SelectKBest, LASSO), and machine learning models were applied to further analyze and classify the metabolite profiles.

**Results:**

Significant differences in phytochemical composition and antioxidant activities were observed among the three onion types. A total of 243 volatile metabolites were detected, with sulfur compounds accounting for 51-64%, followed by organic acids and their derivatives (4-19%). Multivariate analysis revealed distinct volatile profiles, and 19 key metabolites were identified as biomarkers. Additionally, 33 and 38 feature metabolites were selected by SelectKBest and LASSO, respectively. The 38 features selected by LASSO enabled clear differentiation of onion types via PCA, UMAP, and k-means clustering. Among the four machine learning models tested, the random forest model achieved the highest classification accuracy (1.00). SHAP analysis further confirmed 20 metabolites as potential key markers.

**Conclusion:**

The findings suggest that the combination of HS-SPME/GC-MS and machine learning, particularly the random forest algorithm, is a powerful approach for characterizing and classifying volatile metabolite profiles in colored onions. This method holds potential for quality assessment and breeding applications.

## 1 Introduction

Onion (*Allium cepa* L.) is broadly cultivated and consumed for its distinctive flavor and aroma (1). In general, onion germplasms exhibit diverse colors, such as purple or red, white, and yellow colors (2). Red onions are rich in anthocyanins with antioxidant properties, yellow onions have high flavonoid content, particularly quercetin, and white onions contain sulfur compounds with antibacterial effects (3). Studies have shown that pigmented onions provide numerous health benefits, such as anticancer and antibacterial properties, which can be attributed to their bioactive substances, including anthocyanins and flavonoids, as well as various biological activities, such as free radical scavenging ability and phenol concentration (4, 5).

The smell of onions is primarily due to volatile compounds released when the onion is chopped (6). Wang et al. (7) identified 61 volatile odor chemicals (27 sulfur compounds and 13 aldehydes) in several pigmented onions via headspace solid-phase microextraction associated with gas chromatography–mass spectrometry (HS–SPME–GC–MS). D’Auria et al. (8) also identified sulfides (thiopropanal S-oxide) in onions as the key differentiator between onions and shallot via HS-SPME-GC–MS. Li, Q. et al. (9) analyzed purple onions from different origins and reported that the purple onion from Gansu Province is superior in flavor quality, containing 27 sulfides. Therefore, varieties, odorants, colors and geographic position are closely related to the quality characteristics of onions.

Recently, metabolomics has become a highly interdisciplinary and emerging technique that can systematically detect low-molecular-weight metabolites in biological systems (10, 11). Gas chromatography–mass spectrometry (GC–MS) and liquid chromatography–mass spectrometry (LC–MS) are the main analytical techniques used for metabolomics. Compared with LC–MS-based metabolomics, GC–MS-based metabolomics has superior precision, reproducibility, and measurement sensitivity, is widely used and can achieve qualitative evaluation of volatile components. When combined with the HS/SPME procedure, GC–MS-based metabolomics has been confirmed to be a powerful means for quantifying volatile metabolites in various agricultural products. For example, Wang et al. (12) detected 132 volatile metabolites of fermented Kombucha via HS-SPME/GC–MS metabolomics and then selected 25 characteristic metabolites as biomarkers through multivariate statistical analysis. Zhao et al. (13) identify the nontargeted metabolomics of 27 japonica rice varieties from distinct areas of China.

Moreover, 16 kinds of volatiles and 22 kinds of aromatic compounds were selected as discrimination identifiers for uncooked and steamed rice, respectively. Additionally, employing the HS-SPME/GC–QMS technique alongside multivariate statistical analysis has proven to be an effective method for distinguishing the geographical origins of Spanish onion varieties (14). Thus, GC–MS combined with metabolomics is a powerful means to explore characteristic metabolites in onion, providing detailed information on the volatile metabolites that define flavor and aroma.

However, HS-SPME-GC–MS-based metabolomic studies can acquire vast amounts of useful data, which are often processed through multivariable statistics and may contain redundant information. With the rapid development of artificial intelligence, machine learning technologies (such as XG Boost, logistic regression, random forest, and decision tree) have been applied to metabolomic studies. These methods can significantly address these shortcomings and demonstrate great application prospects. In addition, metabolomics employing machine learning (ML) provides an unambiguous perspective on data excavation, visualization, and selection of essential metabolite elements (15). Wu et al. (16) used UPLC–MS/MS to extract metabolites from 32 out of 366 *Astragalus* samples. They employed machine learning algorithms to assess the accuracy of four selected feature recognizers, achieving an accuracy of 86.9%. A combined approach of machine learning and metabolomics can be used to evaluate the role of volatile metabolites in maintaining the freshness of strawberries during storage (17). Therefore, the use of HS-SPME/GC–MS technology in combination with metabolomics and machine learning methods can be a novel approach for identifying the characteristic metabolites of colored onions.

Jiuquan is located in Gansu Province, Western China, which is a famous onion production area and is well known for its three-colored onions (red, white, and yellow) as a National Geographical Indication Product (7). Pigmented onions contain distinct aroma, abundant health-promoting compounds and huge market value for exploitation, Our previous study identified volatile organic compounds in raw and cooked pigmented onion through GC-IMS (6). To date, characterization of volatile flavor profiles of different varieties of onion (18), different geographical regions (14), and various processing methods (19) derived from the traditional HS-SPME-GC–MS approach has been reported. Although previous studies have analyzed volatile compounds in onions, few reports have integrated HS-SPME-GC–MS-based metabolomics with machine learning to classify and identify key metabolites in pigmented onions.

Given their diverse phytochemical profiles and economic importance, this study, on one hand, compared the differences in major nutrients and antioxidant activities of colored onion (red, white, and yellow) cultivated in Jiuquan. On another hand, the volatile metabolites in different pigmented onions were further investigated through HS-SPME-GC–MS-based metabolomics. The distinctive volatile metabolites were also screened via multivariate statistics. Moreover, machine learning approaches were employed to extract the key features of the distinctive volatile metabolites and to evaluate their ability to discriminate pigmented onions. These findings may shed additional light on the quality characteristics of pigmented onions in Jiuquan.

## 2 Materials and methods

### 2.1 Material and reagents

Three colored onions and variety names—Baibilong (white), Hongyou 1 (red), and Jinke 7 (yellow)—were collected on October, 2024 at the same geographical location from Suzhou (Jiuquan, China) (Supplementary Figure S1). The three peeled tinctorial onions were stored at 4°C.

The carotenoid test kit was purchased from Beijing Leagene Biotechnology Co., Ltd. Kits for total antioxidant ability assays (DPPH and ABTS), as well as phenol, flavonoid, and proanthocyanidin test kits, were obtained from Jiancheng Bioengineering Research Institute (Nanjing, China). Anhydrous methanol and ethanol were directly sourced from Tianjin Fuyu Fine Chemical Co., Ltd.

### 2.2 Evaluation of major nutrients and antioxidant activity

The determination of major nutrients (phenols, flavonoids, anthocyanins, and carotenoids) in pigmented onions was carried out according to the methods of Hu et al. (20). Briefly, 0.1 g of each onion mixture was accurately weighed, 1 mL of extraction solution was added according to the kit’s instructions, and the mixture was mixed in a chilled water bath (DZKW-S-4, Shanghai Keheng Industrial Development Co., Ltd., Shaanxi Province, China). Then, the samples were centrifuged at 12,000 RPM (Allegra X-30R, Beckman Coulter, Inc., USA) for 10 min at 4°C, and the supernatants were placed on ice for testing. The total phenol content (TPC), total flavonoid content (TFC), total anthocyanin content (TAC), and carotenoid content were measured via an enzyme-linked assay (Evolution 201, Thermo Fisher Scientific Inc., USA) according to the instructions of the corresponding kits. The antioxidant activity of each onion was also evaluated through DPPH and ABTS radical scavenging rates according to the instructions of the corresponding determination kits. All procedures were repeated three times for each onion sample as biological replicates.

### 2.3 Semiquantitative analysis of volatile metabolites via HS–SPME combined with GC–MS

Each onion sample was ground evenly, precisely weighed (1.0 g), and placed into 20 mL SPME containers manufactured by Shendi in China. Three colors of onions independently were set up in eight parallel groups to ensure the scientific data (n = 8). The GC–MS conditions used in this study followed the methodology of Wei et al. (21), with appropriate adjustments. Onion samples were analyzed via the CTC Triad automatic sampler. SPME was used for 15 min at 50 degrees Celsius and a vibration speed of 250 rpm. The SPME filament was subsequently placed in the vapor phase of the onion samples, allowing them to take up the volatile ingredients for 30 min. After these steps, the filament was placed into the GC injection port for desorption of analytes over 5 min. The cycle time of GC was 50 min, and all samples were analyzed in triplicate. Analysis was performed via an Agilent 7890B-5977B GC–MS system equipped with a DB–WAX capillary column (30 m in length, 0.25 mm internal diameter, 0.25 μm film thickness). The carrier gas used was ultrahigh-purity helium, which flows at a rate of 1.0 mL per min. The preliminary thermic conditions of the column were set at 40°C for 5 min. The temperature was then increased to 220°C at a rate of 5°C/min, followed by an increase to 250°C at a rate of 20°C/min, after which it was held at this final temperature for 2.5 min. The injection degree and interface degree were both set at 260°C. The ion generator and quadrupole thermal conditions were 230°C and 150°C, respectively. The mass spectrometry data were acquired in full-scan mode across a m/z range of 22–400, with the mass selective detector operating in electron impact ionization mode at 70 eV. Using 1,2-dichlorobenzene as the internal standard, the raw data were analyzed by the software ChromaTOF via the total peak normalization correction method to obtain the peak area information of the substances, which represents the quantitative results. The spectra were matched against the NIST2017 spectrum library.

### 2.4 Screening of differentially abundant metabolites and feature metabolites

#### 2.4.1 Multivariate statistical analysis

The datasets of peak areas were scaled via the Pareto method before performing multivariate analysis with principal component analysis (PCA) and orthogonal projections to latent structures discriminant analysis (OPLS-DA). Precise classification models were established via unsupervised PCA and supervised OPLS-DA. The variable importance in projection (VIP) selection method was applied to identify potential markers for colored onions by determining the key compounds that contribute to the separation of each sample in the OPLS-DA score plots. The OPLS-DA models were validated via permutation tests (13).

#### 2.4.2 Building and assessing the machine learning model

Two feature selection methods were used to decrease the feature count, prevent dimensional problems, make the model easier to interpret, and reduce overfitting (22). First, the correlation values among features were calculated, and features exhibiting high multicollinearity (greater than 0.8) were subsequently removed, reducing the feature count from 242 to 167. Additionally, SelectKBest with the function chi2 was used to select key feature metabolites. Second, the least absolute shrinkage and selection operator (LASSO) method was also used to select key feature metabolites. The descriptors were then scaled via the Zheng et al. (22) equation. This scaling ensured that each dimension’s data had a variance of one and a mean of zero. These processed descriptors *x_1_*, *x_2_*, *x_3_*,…, and *x_38_* were subsequently employed as sample variables *X* for regression and classification models (23).

To further enhance the classification and feature selection, machine learning algorithms, including XG Boost, logistic regression, random forest, and decision tree, were employed. XG Boost was chosen for its superior ability to handle complex data interactions and improve classification accuracy through boosting. Logistic regression provided a simple yet effective baseline model for understanding the relationship between metabolites and onion types. Random forest was applied for its robustness in high-dimensional data and its ability to mitigate overfitting. Finally, the decision tree algorithm contributed by offering an interpretable framework for classifying metabolites. These combined methods significantly improved feature selection and model performance in distinguishing pigmented onion varieties.

### 2.5 Statistical analyses

The data are expressed as the means ± standard deviations (n = 8). The possible metabolite names and their retention times, CAS numbers, relevant content, and other information in each sample were obtained through database annotation, and this annotation information of each sample was integrated to obtain the final table of possible metabolites for analysis. The peak data were normalized to analyze the levels of different metabolites. To study the accumulation of metabolites, various analyses, including PCA, OPLS-DA, heatmaps and box plots, UpSet plots, and K-means clustering, were conducted via the R package.^1^ All machine learning analyses are implemented on the Anaconda3 platform, including scikit-learn libraries, glmnet (for LASSO), and matplotlib for data processing and analysis (23).

## 3 Results and discussion

### 3.1 Major nutrients and antioxidant activity of pigmented onion bulbs

The present study investigated the relative contents of major nutrients in pigmented onion. Anthocyanins are related to the color of onions and are found mainly in the outer layers of the onion (19). As shown in Supplementary Figure S2, TAC levels (5.80 ± 1.4 μg/g) were the highest in red-colored onion (*p* < 0.05), but no marked differences were found between white-colored onion (0.6 ± 0.51 μg/g) and yellow-colored onion (0.55 ± 0.25 μg/g) (*p* > 0.05). TFC levels differed distinctly between yellow-colored onions (0.20 ± 0.02 mg/g) and white-colored onions (0.16 ± 0.02 mg/g) (*p* < 0.05). However, there was no significant difference in the TFC between red-colored onions (0.18 mg/g) and the other two pigmented onions. The TPC value of yellow onions (0.69 ± 0.06 mg GAE/g) was significantly greater than that of other-colored onions (*p* < 0.05). The red-colored onion (0.55 ± 0.08 mg GAE/g) was significantly different from the white-colored onion (0.11 ± 0.03 mg GAE/g) (p < 0.05). The carotenoid content showed the same trend as the anthocyanin content. Supplementary Figure S2 also shows the antioxidant activity of different colored onions by the DPPH and ABTS radical scavenging methods. The DPPH radical scavenging activity paralleled the trend observed with TAC. The antioxidant capacity of red-colored and yellow-colored onions is not significant (*p* > 0.05), and red-colored and yellow-colored onions are significantly different from white-colored onions (*p* < 0.05). These results implied that pigmented onions are abundant resources of anthocyanins, phenols, flavonoids, and carotenoids with increased antioxidative potential. A similar study by Zhang et al. (2) reported that TAC, TFC and TPC were positively correlated with antioxidant activity in pigmented onions from Jiangsu Province.

Different onion varieties contain various bioactive compounds that contribute to their color and antioxidant properties. Red onions are particularly rich in anthocyanins, including cyanidin-3-glucoside, cyanidin-3-laminaribioside, and peonidin derivatives, which are responsible for their red-purple pigmentation (3). They also contain significant amounts of flavonoids, particularly quercetin glycosides such as quercetin-3,4′-diglucoside and quercetin-4′-monoglucoside. Additionally, red onions are a source of phenolic compounds, including ferulic acid, p-coumaric acid, gallic acid, and protocatechuic acid, along with small amounts of carotenoids like *β*-carotene and lutein (24). Yellow onions, on the other hand, are abundant in flavonoids such as quercetin and kaempferol derivatives, with quercetin being the predominant compound (3). Their phenolic composition includes ferulic acid and caffeic acid, both known for their antioxidant activities. Yellow onions also contain lutein and zeaxanthin, which contribute to their characteristic coloration (25). White onions have lower flavonoid content than red and yellow varieties, with small amounts of quercetin and isorhamnetin. Their phenolic content is relatively lower as well, primarily consisting of ferulic acid and p-coumaric acid (26). Carotenoid levels in white onions are also minimal compared to yellow onions (3).

### 3.2 Outline of the metabolic characteristics of pigmented onion

#### 3.2.1 HS-SPME/GC–MS-based metabolomic analysis

**Figure 1:**
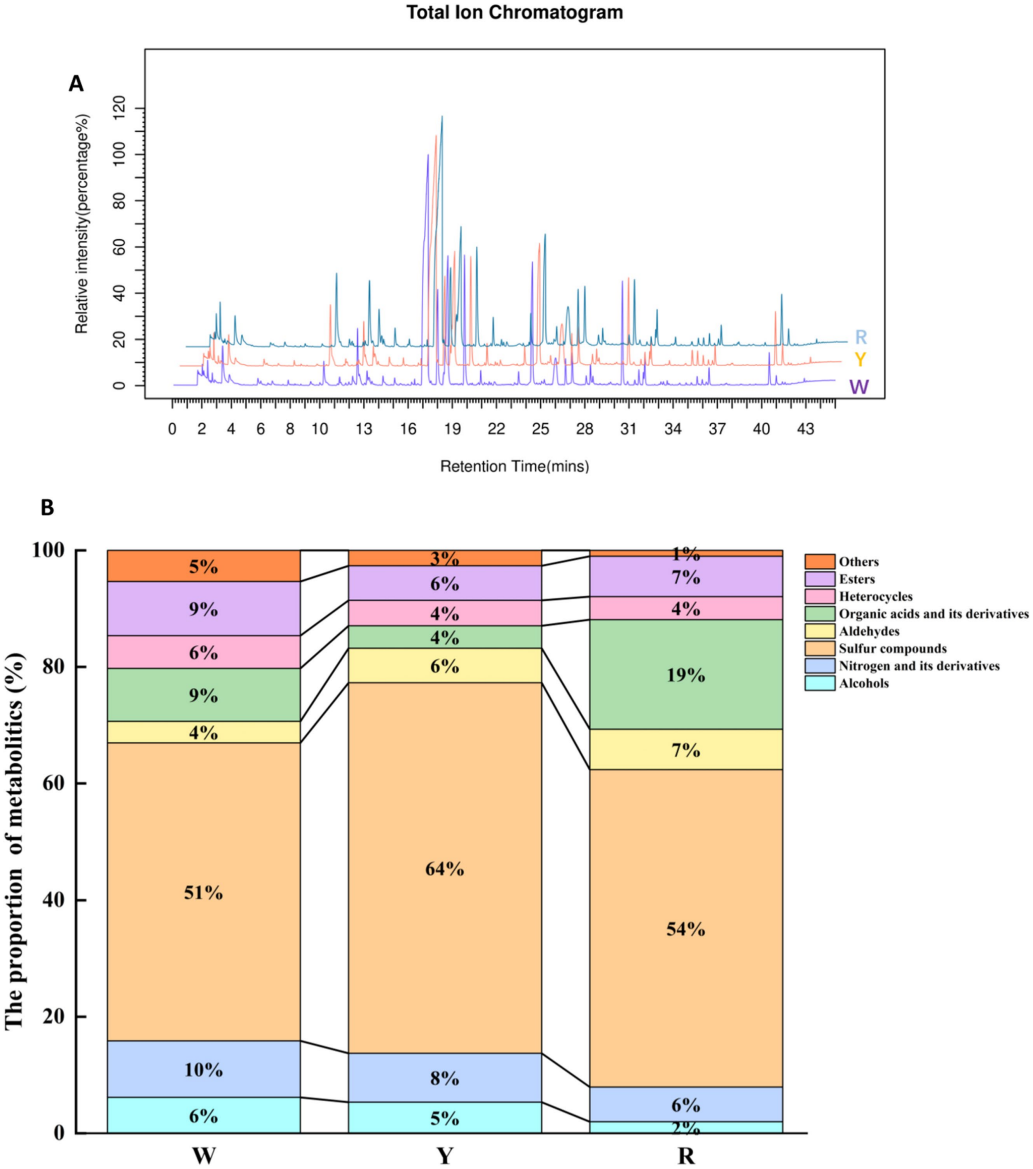
**(A)** Total ion flow diagram of onion samples. **(B)** Distribution of each category of aromatic metabolites in pigmented onion.

The stability of the instrument data collection was verified by comparing the total ion flow diagrams of onion samples for spectral overlap. As depicted in Figure 1A, the baseline of the onion samples remained stable, and the intensity and retention time of each chromatographic peak remained consistent. This indicates reliable instrument stability, with minimal variation due to instrument errors throughout the experiment. To explore the diversity of metabolites, HS-SPME/GCMS was used to analyze and provide details of the volatile metabolites of onions with different colors (9). A total of 243 metabolic species were separated and characterized, which were grouped into alcohols (2–7%), nitrogen and its derivatives (6–10%), sulfur compounds (51–64%), aldehydes (4–7%), organic acids and their derivatives (4–19%), heterocycles (4–6%), esters (6–9%), and others (1–5%). Onion metabolites of different colors were classified, as illustrated in Figure 1B. Sulfur compound levels in white onion were lower than those in yellow and red onion. These sulfur compounds primarily contribute to differences in onion flavor substances, depending on their content and quantity of sulfides. Liu et al. (27) also identified 16 sulfides in freshly chopped onion via HS-SPME/GC–MS and proposed a correlation between volatile sulfur compound fluctuations and various fresh-cut styles and storage temperatures. Moreover, organic acids play an essential role and can influence the flavor profile of onions by influencing their acidity and pH (26). The concentrations of organic acids and related derivatives in different colors significantly varied, specifically red > white > yellow. Das et al. (28) used a GC–MS-dependent metabolomics methodology to detect volatile metabolites in 11 varieties of onion bulbs. Sixty-two metabolites were detected, 11 of which were organic acids.

#### 3.2.2 Multivariate statistical analysis

PCA was used as an unsupervised method to visualize natural clustering and data variability (29). In a study by Fernandes et al. (14), PCA was used to divide Spanish onions from different regions into four distinct clusters: one group consisted of onions from Porto Moniz, another from Ribeira Brava, and two additional groups from Caniço and Santa Cruz, which are located close to each other. Similarly, we employed PCA to discriminate between onion samples of different colors: white vs. yellow, white vs. red, red vs. yellow, and white vs. yellow vs. red, revealing varying degrees of separation. As shown in Supplementary Figure S3, the two major components accounted for 32.4, 34.7, 31.5, and 26.7% of the total variance, respectively. The PCA results revealed discernible differences among the colored onion samples, which were distinguishable through the identification of volatile substances. These findings demonstrated that the onion samples could be distinctly separated, highlighting clear differences in their profiles.

**Figure 2:**
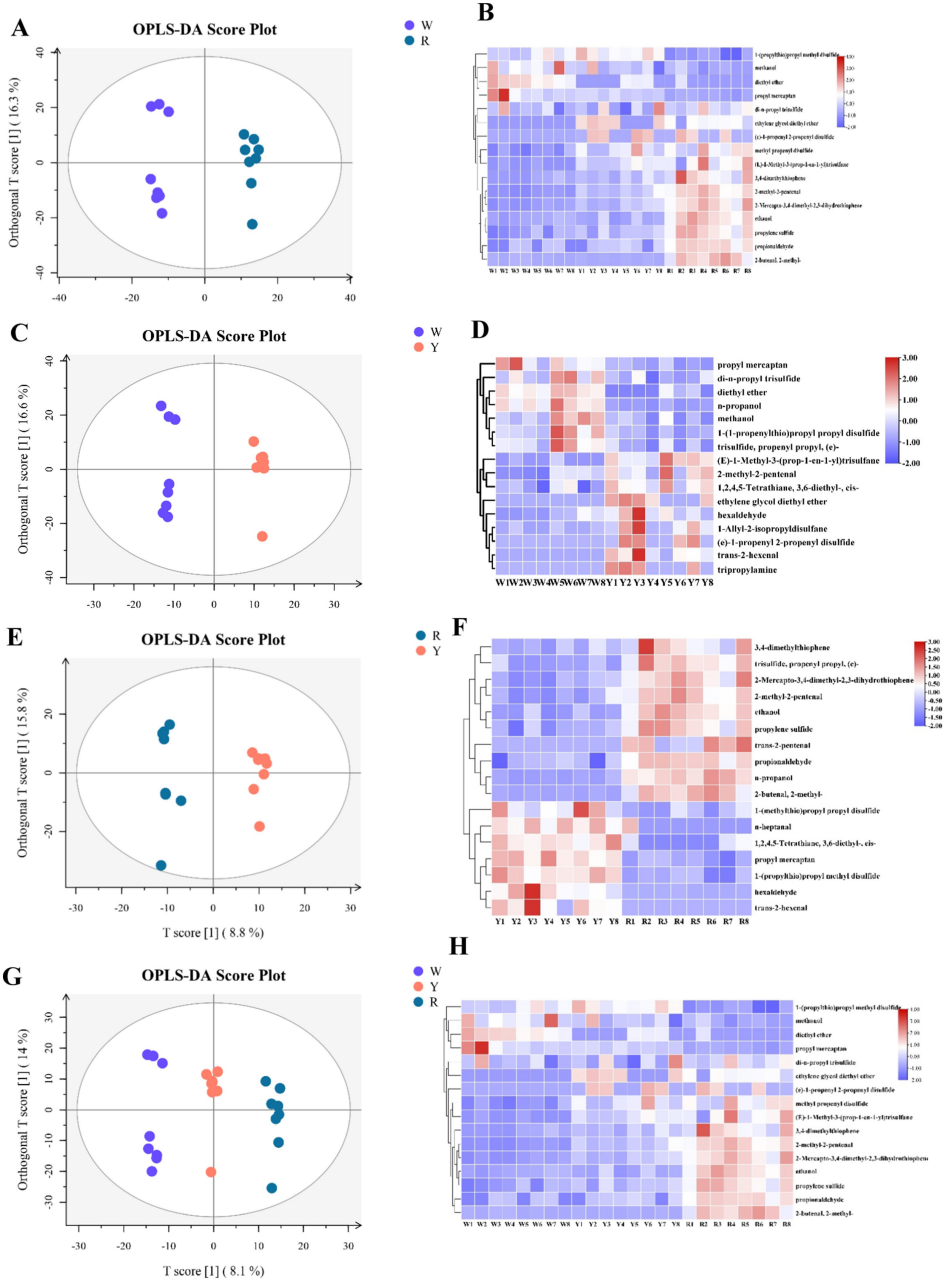
OPLS-DA predictive model and clustered heatmap of significant aromatic metabolites in accordance with VIP scores obtained from three onion colors. **(A,B)** White vs. red, **(C,D)** white vs. yellow, **(E,F)** red vs. yellow, **(G,H)** white vs. yellow vs. red.

In contrast, OPLS-DA served as a supervised model to maximize group separation and identify key discriminative volatile metabolites (30). To determine the differences among the three onion species, OPLS-DA modeling was applied to determine the relative abundance of volatile metabolites (Figures 2A,C,E,G). The validity of the model was subsequently verified through a permutation test. The values of *R^2^Y* and *Q^2^* in the white and yellow, white and red, and red and yellow and white groups were 0.994 and 0.413, 0.988 and 0.547, 0.987 and 0.496, and 0.992 and 0.562, respectively. This shows that the fitting precision of the model is high. The parallel groups of different varieties of onion were clustered together, indicating that the samples in this study were consistent. Briefly, OPLS-DA can distinguish onion groups well and can screen significantly different metabolites.

**Figure 3:**
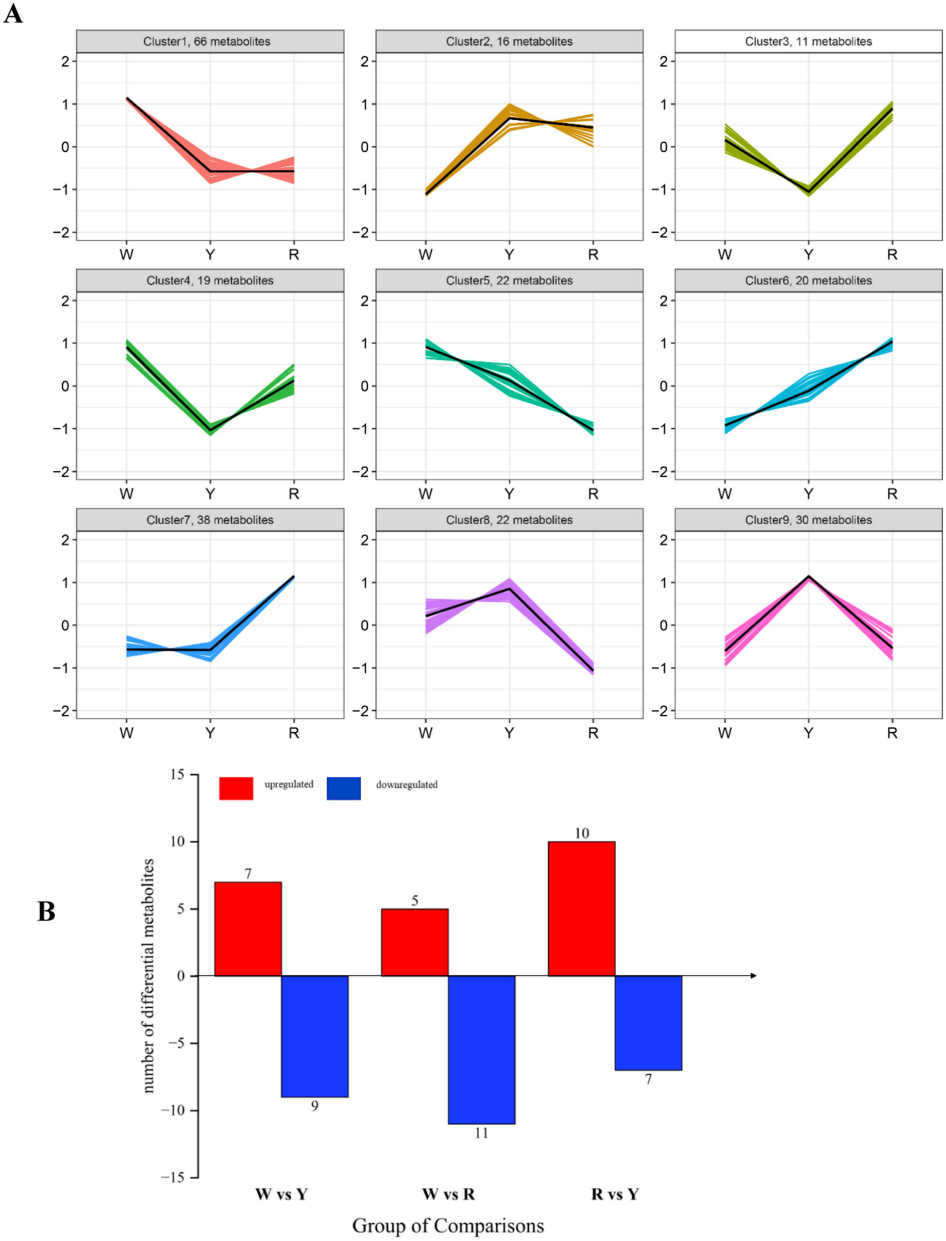
**(A)** K-means examination of various metabolites of three colored onions. The vertical axis displays the normalized quantity of each compound, whereas the horizontal axis represents the various samples. **(B)** Bar chart of the color comparison between the up- and downregulated onion samples.

Using K-means clustering analysis, we filtered the results to obtain 243 differentially abundant metabolites, which were categorized into 9 distinct profiles (Figure 3A). Profiles 1, 4, and 5 revealed that 107 metabolites accumulated more in white onions than in yellow and red onions. These included 17 esters, 15 sulfur compounds, and 13 organic acids and their derivatives. Profiles 2, 8, and 9 included 68 metabolites, mainly 14 esters, 9 organic acids and their derivatives, and 9 sulfur compounds that were present in white and red onions. In profiles 3, 6, and 7, 69 metabolites were overtopped in white and yellow onions, which were principally esters, hydrocarbons, and sulfur compounds. Volatile sulfur compounds contribute to the characteristic odor of onion through the metabolic pathway of S-alkylcysteine sulfoxides (9). The esters, ethyl acetate and propyl acetate, found in onions, originate from the fatty acid and amino acid pathways (27). The present metabonomic analysis suggested that variations in the relative abundance of metabolites, particularly in sulfide- and ester-related pathways (Figure 3A), could account for the flavor differences among white, yellow, and red onions.

### 3.3 Screening and identification of differentially abundant metabolites

Differentially abundant metabolites were identified by screening. The criteria for identifying relevant differentially abundant metabolites included a VIP score >1 and a one-way ANOVA *p* value <0.05. As shown in Figure 3B, 16 differentially abundant metabolites were found in the white- and yellow-colored comparisons, 16 in the white- and red-colored comparisons, and 17 in the red- and yellow-colored comparisons. There were 7, 5 and 10 upregulated metabolites and 9, 11 and 7 downregulated metabolites in white-colored vs. yellow-colored onion, white-colored vs. red-colored onion, and red-colored vs. yellow-colored onion, respectively (Figure 3B).

The metabolite content of white-colored onion was significantly greater than that of yellow-colored onion in 2 alcohols (methanol, 1-propanol), 4 sulfides (dipropyl trisulfide, propyl mercaptan, etc.), and 1 ether. Compared with yellow-colored onion, white-colored onion had a lower content of 3 aldehydes, 4 sulfides (1-allyl-2-isopropyldisulfane, (E)-1-propenyl 2-propenyl disulfide, etc.) tripropylamine and ethylene glycol diethyl ether. The metabolite content of a white-colored onion was significantly greater than that of a red-colored onion for 3 sulfides (di-n-propyl trisulfide, 1-(propylthio) propyl methyl disulfide, and propyl mercaptan), methanol, and diethyl ether. In contrast, the contents of 4 sulfides, 3 aldehydes, 2 thiophenes (3,4-dimethylthiophene, 2-mercapto-3,4-dimethyl-2,3-dihydrothiophene), ethylene glycol diethyl ether, and ethanol were lower in white-colored onions than in red-colored onions. The metabolite content of red-colored onion was greater than that of yellow-colored onion in 2 sulfides ((E)-propenyl propyl, trisulfide, propylene sulfide), 4 aldehydes, 2 thiophenes, and 2 alcohols (ethanol, n-propanol). The contents of 4 sulfides and 3 aldehydes (n-heptanal, hexaldehyde, and trans-2-hexenal) in red onion were lower than those in yellow onion. The white onion (*S. Pietro*) is known for its sweetness and low-pungency odor, and it also has a low content of all the sulfur components (31), which is consistent with the present results (Figure 1B). Compared with darker onions (such as yellow and red onions), aldehydes and sulfur-containing heterocyclic compounds give onions a spicier taste because of differences in their metabolites. This may explain why the levels of aldehydes and sulfur-containing compounds are low in white-colored onions.

**Figure 4:**
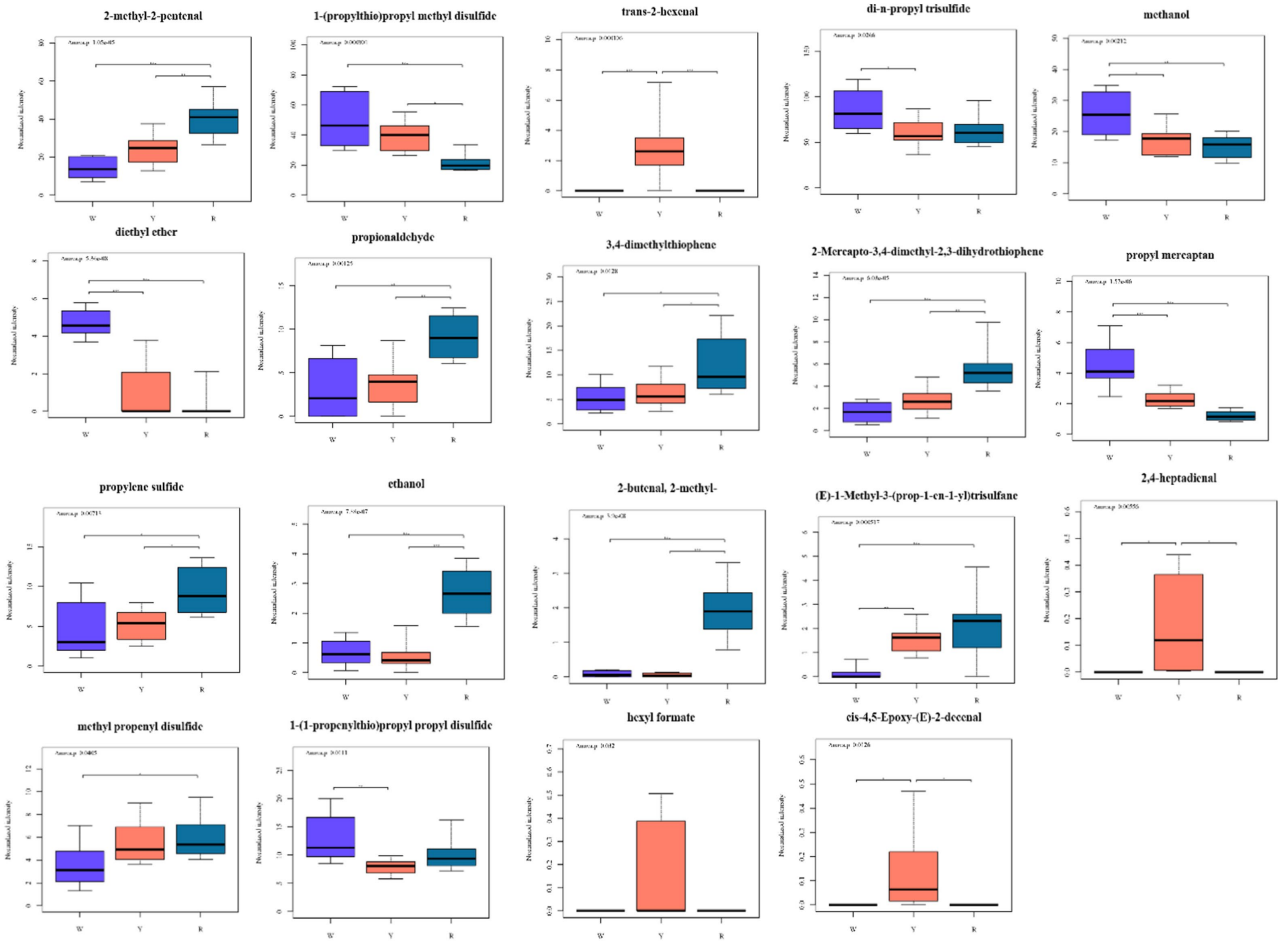
Boxplots of 19 metabolites based on the OPLS-DA (VIP > 1) model showing significant differences between pigmented onions. * denotes a significant difference at the 0.05 level. ** denotes a significant difference at the 0.01 level. *** denotes a significant difference at the 0.001 level.

Afterward, heatmaps were created to visualize the content differences of the potentially characterized metabolites, incorporating dendrograms for enhanced clarity (Figures 2B,D,F,H). These results, which are consistent with the above multivariate analysis, revealed that the key metabolites were categorized into two distinct classes. There were 19 different metabolites in the three groups that contained white-colored, yellow-colored, and red-colored onions. The three groups of different metabolites were characterized via a box plot (Figure 4).

As shown in Figure 4, the 19 different metabolites can be classified as sulfides, aldehydes, ketones, and others. Sulfur compounds are the primary contributors to the distinctive aroma of onions and play a crucial role in defining their scent profile. The differences in odor among species depend on the type and quantity of these sulfur compounds, which shape their volatile metabolites (32). Research has indicated that in onions, S-[(E)-Prop-1-enyl]-L-cysteine S-oxide serves as the primary pioneer of sulfur-containing volatiles. When onions are diced, the enzyme alliinase reacts with S-[(E)-Prop-1-enyl]-L-cysteine S-oxide to produce a sequence of reactive sulfonic acids, which undergo chemical transformations to form stable volatiles, such as mono-, di-, and trisulfides (18). According to Liu et al. (27), propyl mercaptan, dipropyl disulfide, and methyl propyl trisulfide are the key volatile onion compounds. The propyl mercaptan and 1-(1-propenylthio) propyl propyl disulfide of white-colored onion differs significantly from those of yellow-colored onion and red-colored onion (Figure 4). Propylene sulfide has the opposite effect. The 1-(1-propenylthio) propyl propyl disulfide and di-n-propyl trisulfide of yellow-colored onion have the lowest metabolite intensities. The methyl propenyl disulfide of white-colored onions has the lowest metabolite intensity. As a result, these six sulfides can act as significantly different metabolites that distinguish different colored onions. Dipropyl disulfide and dipropyl trisulfide are the main volatile compounds present in fresh onions and are the primary reason for their strong aroma (27). The type and amount of these compounds determine how spicy the onion is. The heat treatment causes a change in the sulfur content between raw and cooked green onions, thus affecting the raw green onions to be more spicy than cooked green onions (33).

Aldehydes and alcohols were the most abundant compounds, resulting in a green aroma reminiscent of the freshly cut green or woody smell of plants. Aliphatic aldehydes and alcohols are produced when plant tissues are cut and chewed and originate from the lipoxygenase pathway. Several aldehydes (short-chain C6 aldehydes) have been identified, which are likely formed from fatty acids through the LOX pathway (34). This pathway is primarily active in the green tissues of plants in response to injury, contributing to the production of volatiles with green fragrance. Cecchi et al. (35) also detected 2-methyl-2-butenal, 2-methyl-2-pentenal, and propionaldehyde in dried onion via HS-SPME-GC–MS, HS-SPME-GC × GC-TOF, and HPLC-DAD. This may be due to the state of the onion sample. The 2-methyl-2-butenal, 2-methyl-2-pentenal, and propionaldehyde contents of red-colored onion are significantly different from those of white-colored and yellow-colored onion. The trans-2-hexenal and 2,4-heptadienal of yellow-colored onion are the most abundant (Figure 4).

Elevated injection temperatures have been implicated in the formation of thiophene compounds (36). Taglienti et al. (32) identified 2-mercapto-3,4-dimethyl-2,3 dihydrothiophene and 2,4-dimethylthiophene. The 2-mercapto-3,4-dimethyl-2,3-dihydrothiophene and 3,4-dimethylthiophene contents of the three colors of onions were significantly different. Red onions have the highest levels, whereas white onions have the lowest. This is similar to previous report, which may provide additional insights into the odor profiles of colored onions both academically and industrially (5).

(E)-1-Methyl-3-(prop-1-en-1-yl) trisulfane, and ethanol are the most abundant red onions. The contents of hexyl formate and cis-4,5-epoxy-(E)-2-decenal in yellow onion are significantly greater than those in white onion and red onion. Diethyl ether and methanol are the most abundant white-colored onions (Figure 4). Esters, which are primarily formed through chemical reactions between acids and alcohols, contribute sweet and fragrant aromas to onions, resulting in differences in the tastes of colored onions (5). Pearson correlation analysis was conducted between 19 differentially abundant metabolites and major nutrients as well as antioxidant activities, and the results are shown in Supplementary Figure S4. The total anthocyanin content was positively correlated with di-n-propyl trisulfide (*p* < 0.05), whereas hexyl formate, cis-4,5-epoxy-(E)-2-decenal, trans-2-hexenal, and 2,4-heptadienal were negatively correlated with ABTS (*p* < 0.01).

### 3.4 Selection of feature metabolites in pigmented onions via machine learning

The 243 identified metabolites exhibited numerous overlapping features, prompting us to employ machine learning models and assess the feasibility of feature selection as well as classification accuracy across three colored onions. Therefore, we preprocessed the data via the LASSO method, and 38 feature metabolites were selected and used for machine learning. To assess the impact of the Lasso method on eigenvalue processing, a dimensionality reduction technique was employed to reduce and visualize 243 metabolites from the original dataset of three types of onions. Among these, 33 metabolites were selected via the multicollinearity-binding Chi method, whereas 38 were chosen via the Lasso method, which is known for its strong anti-overfitting capabilities and insensitivity to collinearity among variables (37). The 38 features selected by the Lasso method effectively distinguished the differences among the three kinds of onions (Supplementary Figure S5). PCA revealed that the first two components cannot explain the overall changes, indicating that there is a nonlinear relationship between these metabolites. The nonlinear dimensionality reduction UMAP method confirms this, and the Lasso treatment is superior to the multicollinearity binding Chi method. The unsupervised learning K-means algorithm shows similar results (Supplementary Figure S6).

Compared with supervised learning, unsupervised learning can help uncover hidden patterns and structures in data without the need for prelabeled labels, which can help understand the relationships between data features and the underlying clustering structure. The K-means clustering algorithm is widely recognized as one of the most frequently utilized and well-known unsupervised learning algorithms (38). In K-means clustering, we aim to partition the samples in the dataset into K clusters such that each sample is assigned to the centroid of the nearest cluster, thereby delineating the distinctions among the samples. Therefore, on the basis of UMAP, we explored the application potential of the unsupervised learning K-means algorithm in distinguishing three kinds of onions and evaluated the effectiveness of LASSO processing in classifying three kinds of onions. Supplementary Figure S6 showed that the K-means algorithm, which is based on the original 243 data points, could not correctly classify the 3 kinds of onion into 3 clusters. In contrast, the K-means algorithm can obtain 38 variables accurately divided into 3 kinds of clusters according to 3 kinds of onion based on the LASSO method, and the error rate is small, which indicates that LASSO feature processing combined with the K-means algorithm can accurately distinguish 3 kinds of onion without manual labeling. McEligot et al. (39) employed LASSO regression techniques to analyze dietary intake in relation to breast cancer, demonstrating that this approach can effectively identify and clarify the impact of various dietary factors on breast cancer diagnosis. Therefore, 38 feature metabolites in pigmented onion were selected via LASSO for the next prediction.

**Figure 5:**
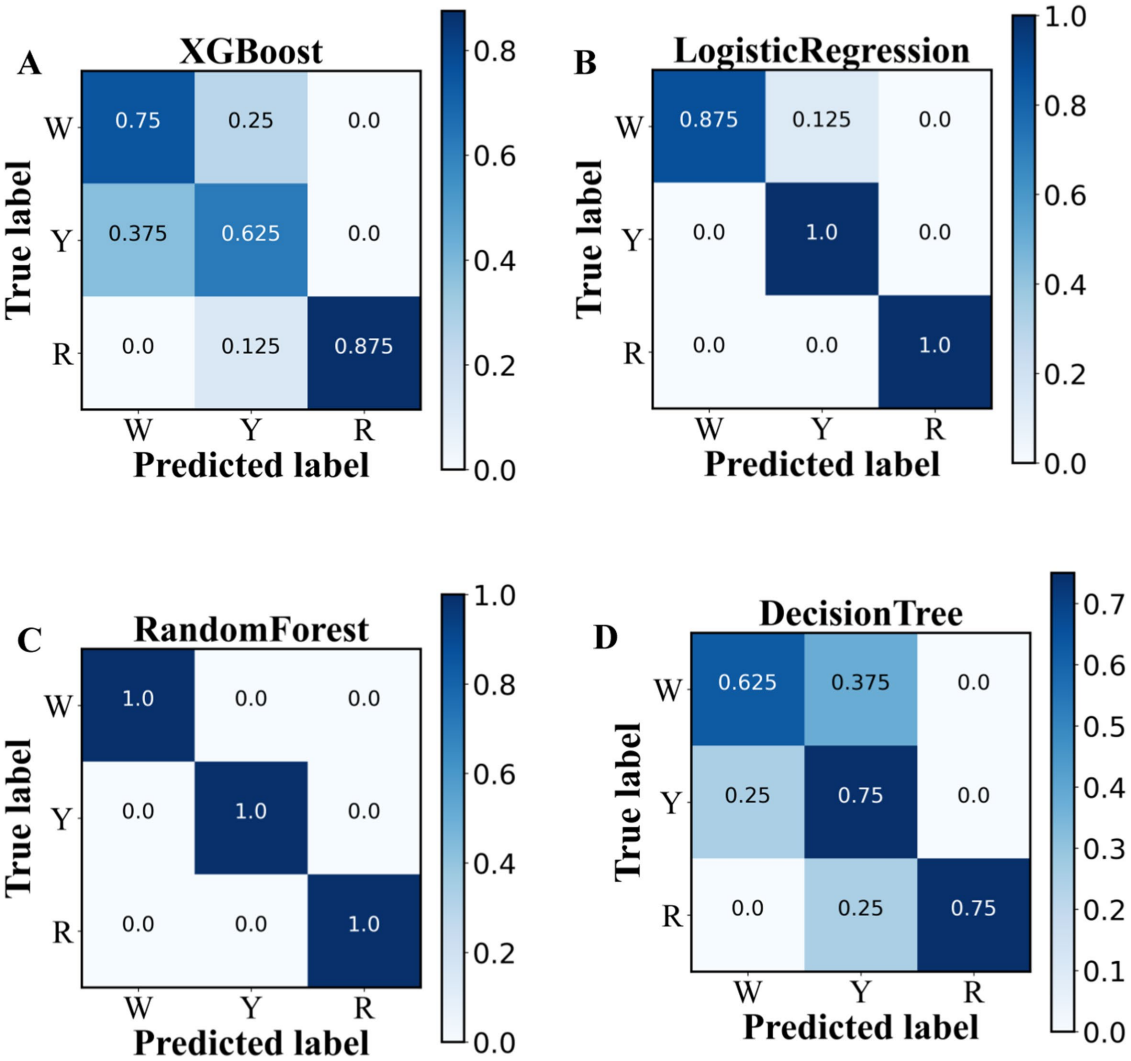
Classification performances of 38 key metabolites in pigmented onions via machine learning algorithms. **(A)** XGBoost, **(B)** logistic regression, **(C)** random forest, and **(D)** decision tree.

For further prediction of the onion characteristic metabolite, four ML (i.e., XG Boost, RF, logistic regression, and decision tree) classification models were constructed and validated via threefold cross-validation, as shown in Supplementary Table S2 and Figures 5A–D. The results indicate that the random forest (RF) model with threefold cross-validation achieved optimum precision, recall, and F1 score above 96% for three colored options, outperforming the performance of the other three models (Supplementary Table S2). This result can be explained by the random forest model introducing randomness, having good noise “immunity” and being appropriate for discrete use (22). To predict the astringency threshold and type of flavonoid compounds, the most effective model was RF, which was analyzed via the Shapley additive explanations (SHAP) approach (23).

### 3.5 Using SHAP analysis to pinpoint essential metabolites as biomarkers

**Figure 6:**
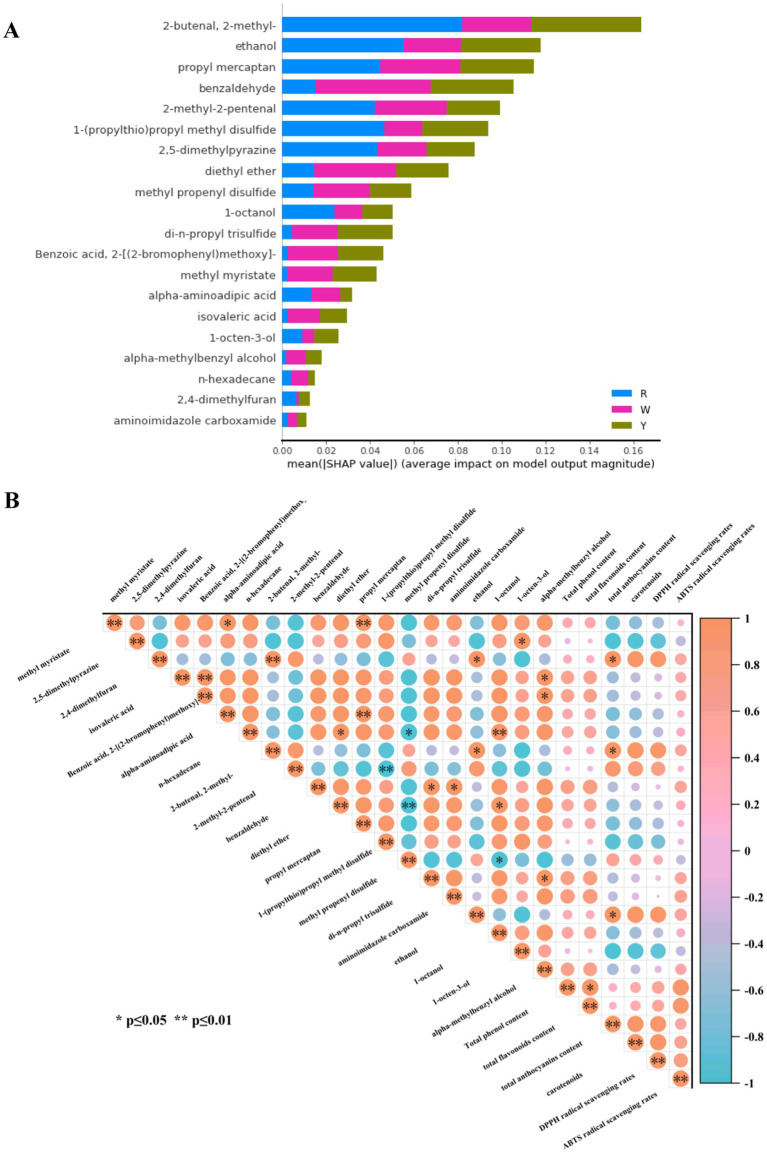
SHAP analysis of the feature metabolites was performed. **(A)** SHAP value distribution of the top 20 compounds as significant indicators of three colored onions. **(B)** Pearson correlation analysis of feature biomarkers, major nutrients, and antioxidant activities in colored onions. * Denotes a significant difference at the 0.05 level. ** denotes a significant difference at the 0.01 level.

The SHAP method has been employed to evaluate the output of the optimum random forest model and pinpoint the features that exert the greatest influence on model predictions (40). Using the SHAP approach, 20 metabolites were further selected from 38 features, as shown in Figure 6A. Moreover, deeper shades of red indicate higher feature values, whereas darker shades of blue signify lower feature values, as depicted in Figure 6A. As shown, 2-methyl-2-butenal, ethanol, and propyl mercaptan contributed more to differentiating the three colored onion groups. Pearson correlation analysis was conducted between 20 selected feature metabolites and major nutrients as well as their antioxidant activities, and the results are shown in Figure 6B. The total anthocyanin content was positively correlated with 2,4-dimethylfuran, 2-methyl-2-butenal, and ethanol contents (*p* < 0.05), as the variation in the content of these substances influences the total anthocyanin content.

We grouped the 20 metabolites (Supplementary Figure S7) and showed that 4 sulfur compounds, 1 diethyl ether, and 3 aldehydes (with added benzaldehyde) were consistent with the 20 differentially abundant metabolites screened by the OPLS-DA model (Figure 4). In addition, there were 3 alcohols (1-octanol, 1-octen-3-ol, and alpha-methylbenzyl alcohol), 3 organic acids and their derivatives (isovaleric acid, 2-[(2-bromophenyl) methoxy]-benzoic acid, and alpha-aminoadipic acid), 2 heterocycles (2,5-dimethylpyrazine, 2,4-dimethylfuran), and others (methyl myristate, n-hexadecane, and aminoimidazole carboxamide), which differed from the two feature selection methods (OPLS-DA and machine learning). Yagin et al. (41) used a hybrid support vector machines + multilayer perceptron model and SHAP to identify glucose, glycine, creatinine, and various phosphatidylcholines as biomarkers for targeted metabolomics analysis of patients with diabetic retinopathy. Ping et al. (42) developed a novel approach for the nondestructive traceability of *Panax ginseng* origin via optimized models that combine hyperspectral imaging (HSI) and X-ray technology. Four variable screening methods were used to optimize the random forest and support vector machine models, which demonstrated high accuracy and potential for tracing medicinal and food products. The machine learning results were similar to those of eight metabolites identified by OPLS-DA, including four sulfur compounds, two aldehydes, diethyl ether, and ethanol. Although the majority of differentially abundant metabolites used as biomarkers were screened by OPLS-DA combined with VIP scores, this study compared the differentially abundant metabolites and feature metabolites sieved by OPLS-DA and machine learning, respectively. The two methods can be used together to screen key metabolites comprehensively and provide samples with detailed information, especially in large datasets of metabolomic studies.

## 4 Conclusion

In summary, red-colored onions contained the most abundant anthocyanins and carotenoids, while yellow-colored onions presented the highest levels of phenols and flavonoids. Both red-colored and yellow-colored onions exhibited the highest DPPH and ABTS radical scavenging activities, highlighting their strong antioxidant potential. A total of 243 volatile metabolites were identified across different colored onions, with sulfur compounds (51–64%), organic acids and their derivatives (4–19%), and nitrogen compounds and their derivatives (6–10%) as the major categories. Sulfur compounds were most abundant in yellow-colored onions, followed by red-colored and white-colored onions. A total of 19 differentially abundant metabolites were identified through multivariate statistical analysis (OPLS-DA model and VIP values). Additionally, using the LASSO method on 243 metabolites, 38 were found to better represent the overall metabolite profiles of the three onion varieties. These 38 metabolites were then applied in four machine learning models for onion classification, with the random forest model achieving perfect classification accuracy (1.00). Furthermore, SHAP analysis identified 20 metabolites as potential biomarkers. Understanding the metabolite composition and antioxidant properties of different onion varieties can aid in selecting and developing onions with enhanced nutritional and sensory attributes. Moreover, the identification of key biomarkers and the successful application of machine learning models provide valuable tools for the classification and authentication of onion varieties in agricultural and food industries. These findings would provide insights into onion breeding, quality control, and authentication of colored onions. More work on expanded sample size, model validation, and uncovering the metabolic pathways influencing onion quality and flavor will be carried out in future.

## Data Availability

The original contributions presented in the study are included in the article/Supplementary material, further inquiries can be directed to the corresponding authors.

## Footnotes

^1^ https://www.r-project.org
