# Supplementary material for: Unraveling volatile metabolites in pigmented onion (Allium cepa L.) bulbs through HS-SPME/GC–MS-based metabolomics and machine learning
Source: Front Nutr. 2025 Apr 22;12:1582576. doi: 10.3389/fnut.2025.1582576 (PMC12052541; doi:10.3389/fnut.2025.1582576)
Supplement: Supplementary file 1 [file Table_1.docx]

Supplementary Material

| **Table S1** A total of 390 volatile metabolites and their classes identified in three colored onions through HS-SPME-GC-MS based metabolomics | | | | | | |
| --- | --- | --- | --- | --- | --- | --- |
| Number | Name | RT | CAS | W | Y | R |
|  | **sulfur compounds (43)** |  |  |  |  |  |
| 1 | n-propyl disulfide | 17.359 | 629-19-6 | 132.126 | 283.201 | 237.421 |
| 2 | 1-allyl-2-isopropyldisulfane | 18.420 | 67421-85-6 | 0.658 | 3.233 | 18.217 |
| 3 | di-n-propyl trisulfide | 24.439 | 6028-61-1 | 85.583 | 60.695 | 62.485 |
| 4 | methyl mercaptan | 1.992 | 74-93-1 | 0.431 | 0.141 | 0.163 |
| 5 | propyl mercaptan | 2.709 | 107-03-9 | 4.531 | 2.286 | 1.207 |
| 6 | 2-thiapentane | 3.674 | 3877-15-4 | 0.246 | 0.005 | 0.006 |
| 7 | propylene sulfide | 3.878 | 1072-43-1 | 4.693 | 5.194 | 9.474 |
| 8 | 3-Propylthio-1-propene | 9.410 | 27817-67-0 | 1.903 | 1.429 | 1.647 |
| 9 | propanethial s-oxide | 12.240 | 32157-29-2 | 3.126 | 3.311 | 2.618 |
| 10 | methyl propyl disulfide | 12.570 | 2179-60-4 | 22.304 | 21.055 | 22.645 |
| 11 | methyl propenyl disulfide | 14.282 | 23838-19-9 | 3.539 | 5.552 | 5.963 |
| 12 | methyl propyl trisulfide | 20.932 | 17619-36-2 | 5.566 | 5.675 | 7.629 |
| 13 | (E)-Prop-1-en-1-yl propanedithioate | 21.640 | 67269-06-1 | 0.231 | 5.05×10^-6^ | 5.05×10^-6^ |
| 14 | 4-hydroxypropranolol sulfate ester | 21.827 | 87075-33-0 | 0.507 | 5.05×10^-6^ | 5.05×10^-6^ |
| 15 | dazomet | 24.335 | 533-74-4 | 2.253 | 1.274 | 1.717 |
| 16 | allyl propyl trisulfide | 25.721 | 33922-73-5 | 0.172 | 0.341 | 0.587 |
| 17 | (E)-trisulfide, propenyl propyl | 26.925 | 23838-27-9 | 19.758 | 12.161 | 18.919 |
| 18 | 1-(propylthio)propyl methyl disulfide | 30.563 | 126876-21-9 | 49.868 | 39.142 | 21.415 |
| 19 | 1-(methylthio)propyl propyl disulfide | 28.383 | 126876-22-0 | 5.688 | 6.846 | 3.583 |
| 20 | 1-(1-propenylthio) propyl propyl disulfide | 32.066 | 143193-11-7 | 12.994 | 7.876 | 10.065 |
| 21 | methyl 1-(1-propenylthio) propyl disulfide | 33.157 | 126876-23-1 | 0.883 | 0.378 | 0.702 |
| 22 | 6-ethyl-4,5,7,8-tetrathianonane | 34.439 | 126876-30-0 | 1.115 | 0.92 | 1.327 |
| 23 | trans-3,6-diethyl-1,2,4,5-tetrathiane | 34.872 | 934273-77-5 | 1.642 | 0.817 | 1.562 |
| 24 | cis-3,6-diethyl-1,2,4,5-Tetrathiane | 35.230 | 934273-79-7 | 1.572 | 3.449 | 0.867 |
| 25 | 6-ethyl-4,5,7,8-tetrathiaundecane | 36.425 | 126876-35-5 | 7.248 | 5.534 | 5.709 |
| 26 | (E)- 1-(1-propenyldithio) propyl propyl disulfide | 37.538 | 126876-36-6 | 0.474 | 0.531b | 0.688 |
| 27 | diisopropyl disulfide | 15.199 | 4253-89-8 | 0.378 | 0.633 | 1.091 |
| 28 | ethiolate | 18.633 | 2941-55-1 | 8.966 | 5.05×10^-6^ | 5.05×10^-6^ |
| 29 | dimethyl disulfide | 7.489 | 624-92-0 | 0.151 | 0.154 | 0.193 |
| 30 | propyl sulfide | 7.516 | 111-47-7 | 0.205 | 0.151 | 0.173 |
| 31 | butyl propyl disulfide | 19.602 | 72437-64-0 | 0.514 | 0.395 | 0.432 |
| 32 | (Z)- 1-(1-propenyldithio) propyl propyl disulfide | 37.421 | 126876-37-7 | 0.124 | 0.027 | 0.223 |
| 33 | (Z)-methyl propenyl disulfide | 13.517 | 23838-18-8 | 0.136 | 5.05×10^-6^ | 5.05×10^-6^ |
| 34 | methyl 1-(methylthio)propyl disulfide | 25.560 | 53897-66-8 | 0.116 | 0.211 | 0.057 |
| 35 | 1-methylpropyl propyl disulfide | 19.602 | 59849-54-6 | 5.05×10^-6^ | 0.111 | 0.056 |
| 36 | dichlorfop-methyl | 29.708 | 51338-27-3 | 5.05×10^-6^ | 0.276 | 0.014 |
| 37 | di-1-propenyl sulfide | 12.224 | 33922-80-4 | 0.053 | 0.031 | 0.087 |
| 38 | (E)-1-propenyl 2-propenyl disulfide | 19.343 | 122156-02-9 | 5.05×10^-6^ | 3.694 | 2.956 |
| 39 | (E, E)-di-1-propenyl disulfide | 19.232 | 23838-23-5 | 5.05×10^-6^ | 0.018 | 0.332 |
| 40 | 2-thioacetyl mage | 2.413 | 112014-15-0 | 0.056 | 0.071 | 1.859 |
| 41 | thiram | 24.304 | 137-26-8 | 5.05×10^-6^ | 5.05×10^-6^ | 0.119 |
| 42 | 1,2-dithiole | 20.766 | 288-26-6 | 5.05×10^-6^ | 0.268 | 0.154 |
| 43 | allyl methyl disulfide | 14.052 | 2179-58-0 | 0.037 | 0.097b | 0.128 |
|  | **amino acids and its derivatives (32)** |  |  |  |  |  |
| 44 | 2-methylbutyrylcarnitine | 12.809 | 256928-75-3 | 0.018 | 5.05×10^-6^ | 5.05×10^-6^ |
| 45 | Phe-Lys | 18.637 | 0-00-0 | 6.835 | 5.05×10^-6^ | 5.05×10^-6^ |
| 46 | nsc337715 | 10.349 | 39217-38-4 | 5.05×10^-6^ | 5.05×10^-6^ | 1.635 |
| 47 | sarcosine | 26.047 | 107-97-1 | 2.166 | 3.569 | 0.905 |
| 48 | glutamyl isoleucine | 2.913 | 5879-22-1 | 0.27 | 5.05×10^-6^ | 5.05×10^-6^ |
| 49 | aceturic acid | 17.351 | 543-24-8 | 38.079 | 36.01 | 30.271 |
| 50 | alanylproline | 17.937 | 13485-59-1 | 2.001 | 0.971 | 0.798 |
| 51 | n-acetyl-s-farnesylcysteine | 2.144 | 135304-07-3 | 1.86 | 2.314 | 5.05×10^-6^ |
| 52 | albizziin | 24.330 | 1483-07-4 | 0.841 | 0.25 | 5.05×10^-6^ |
| 53 | alanylglycine | 26.034 | 687-69-4 | 4.496 | 5.616 | 3.222 |
| 54 | 4-chlorophenylurea | 30.454 | 140-38-5 | 0.021 | 0.011 | 5.05×10^-6^ |
| 55 | d-pyroglutamic acid | 2.948 | 4042-36-8 | 0.128 | 5.05×10^-6^ | 5.05×10^-6^ |
| 56 | dl-homocysteine | 24.339 | 454-29-5 | 0.592 | 0.745 | 5.05×10^-6^ |
| 57 | l-valyl-l-serine | 30.550 | 13588-94-8 | 4.319 | 5.05×10^-6^ | 5.05×10^-6^ |
| 58 | l-glycylalanine | 28.337 | 3695-73-6 | 0.09 | 0.176 | 0.094 |
| 59 | l-cysteinesulfinic acid | 28.329 | 1115-65-7 | 0.02 | 5.05×10^-6^ | 0.001 |
| 60 | s-methylcysteine | 18.568 | 1187-84-4 | 5.05×10^-6^ | 4.134 | 1.763 |
| 61 | n-acetylvaline | 16.925 | 3067-19-4 | 5.05×10^-6^ | 0.132 | 5.05×10^-6^ |
| 62 | gamma-glutamyl-s-allylcysteine | 24.339 | 91216-95-4 | 0.031 | 0.468 | 5.05×10^-6^ |
| 63 | 3-(R)-amino-2-(S)-hydroxy-4-phenylbutanoyl-(S)-leucine | 1.738 | 58970-76-6 | 5.05×10^-6^ | 0.297 | 0.466 |
| 64 | captopril | 24.365 | 62571-86-2 | 5.05×10^-6^ | 1.876 | 5.05×10^-6^ |
| 65 | l-methionylglycine | 24.452 | 14486-03-4 | 5.05×10^-6^ | 0.698 | 5.05×10^-6^ |
| 66 | d-ornithine | 17.268 | 348-66-3 | 5.05×10^-6^ | 20.002 | 5.05×10^-6^ |
| 67 | oxalylglycine | 24.339 | 5262-39-5 | 5.05×10^-6^ | 0.495 | 0.596 |
| 68 | 3-hydroxymethylglutaric acid | 30.976 | 503-49-1 | 5.05×10^-6^ | 0.017 | 0.041 |
| 69 | 3-amino-4-phenylbutanoic acid | 29.706 | 15099-85-1 | 5.05×10^-6^ | 5.05×10^-6^ | 0.017 |
| 70 | n-tert-butyloxycarbonyl-l-valine | 30.450 | 13734-41-3 | 0.003 | 5.05×10^-6^ | 0.004 |
| 71 | prolylglutamic acid | 25.999 | 67644-00-2 | 5.05×10^-6^ | 5.05×10^-6^ | 7.052 |
| 72 | n-(2-amino-1-hydroxypropylidene)-beta-alanine | 26.130 | 52788-02-0 | 5.05×10^-6^ | 5.05×10^-6^ | 0.662 |
| 73 | His-His | 19.858 | 0-00-0 | 18.12 | 7.795 | 9.748 |
| 74 | dl-ethionine | 19.813 | 67-21-0 | 22.11 | 13.801 | 7.67 |
| 75 | phosphoserine | 26.957 | 17885-08-4 | 5.05×10^-6^ | 4.66×10^-04^ | 5.05×10^-6^ |
|  | **Alcohols (26)** |  |  |  |  |  |
| 76 | methanol | 3.413 | 67-56-1 | 25.806 | 17.113 | 15.137 |
| 77 | ethanol | 4.045 | 64-17-5 | 0.684 | 0.552 | 2.696 |
| 78 | 2-propyl-1-heptanol | 12.124 | 10042-59-8 | 0.107 | 5.05×10^-6^ | 5.05×10^-6^ |
| 79 | n-propanol | 6.788 | 71-23-8 | 3.726 | 1.098 | 2.771 |
| 80 | 1-hexanol | 16.929 | 111-27-3 | 6.27 | 9.397 | 1.919 |
| 81 | (E)- 2-methyl-2-Penten-1-ol | 17.698 | 16958-19-3 | 0.085 | 5.05×10^-6^ | 0.145 |
| 82 | 1-octen-3-ol | 19.146 | 3391-86-4 | 0.201 | 0.177 | 0.068 |
| 83 | 1-octanol | 21.914 | 111-87-5 | 1.136 | 0.385 | 0.178 |
| 84 | 2-chloro-5-nitrobenzyl alcohol | 29.615 | 80866-80-4 | 0.032 | 5.05×10^-6^ | 5.05×10^-6^ |
| 85 | 2-phenylethanol | 29.706 | 60-12-8 | 0.456 | 5.05×10^-6^ | 5.05×10^-6^ |
| 86 | 2,3-butanediol | 2.005 | 513-85-9 | 0.14 | 5.05×10^-6^ | 5.05×10^-6^ |
| 87 | n-pentyl alcohol | 13.576 | 71-41-0 | 0.379 | 0.821 | 5.05×10^-6^ |
| 88 | 3-pentanol | 15.997 | 584-02-1 | 0.245 | 0.078 | 0.127 |
| 89 | 2-methyl-1-pentanol | 16.938 | 105-30-6 | 0.989 | 5.05×10^-6^ | 0.2 |
| 90 | 1,4-dihydroxy-2-butene | 18.685 | 110-64-5 | 8.559 | 5.05×10^-6^ | 5.05×10^-6^ |
| 91 | alpha-methylbenzyl alcohol | 29.706 | 98-85-1 | 0.375 | 5.05×10^-6^ | 5.05×10^-6^ |
| 92 | 2-decanol | 22.844 | 1120-06-5 | 0.129 | 0.082 | 0.095 |
| 93 | 2-[methyl(piperidin-4-yl) amino] ethan-1-ol | 6.442 | 864710-80-5 | 0.011 | 5.05×10^-6^ | 5.05×10^-6^ |
| 94 | 4-methyl-2-pentanol | 16.951 | 108-11-2 | 0.649 | 0.71 | 0.215 |
| 95 | cathine | 30.558 | 492-39-7 | 0.006 | 4.609 | 5.05×10^-6^ |
| 96 | ambroxol | 28.403 | 18683-91-5 | 5.05×10^-6^ | 1.503 | 5.05×10^-6^ |
| 97 | 2-propyl-1-pentanol | 30.524 | 58175-57-8 | 5.05×10^-6^ | 5.526 | 5.05×10^-6^ |
| 98 | 1-nonanol | 16.638 | 143-08-8 | 5.05×10^-6^ | 0.05 | 0.008 |
| 99 | fucitol | 25.160 | 13074-06-1 | 5.05×10^-6^ | 0.034 | 5.05×10^-6^ |
| 100 | (10)-gingerol | 28.085 | 23513-15-7 | 5.05×10^-6^ | 5.05×10^-6^ | 0.323 |
| 101 | E-11,13-Tetradecadien-1-ol | 30.945 | 0-00-0 | 0.03 | 5.05×10^-6^ | 5.05×10^-6^ |
|  | **Aldehydes (29)** |  |  |  |  |  |
| 102 | hexaldehyde | 7.859 | 66-25-1 | 1.535 | 3.812 | 0.299 |
| 103 | 2-methyl-2-pentenal | 10.297 | 623-36-9 | 14.226 | 24.01 | 40.103 |
| 104 | n-heptanal | 11.196 | 111-71-7 | 0.614 | 0.993 | 0.103 |
| 105 | glutaraldehyde | 16.944 | 111-30-8 | 0.317 | 0.931 | 0.789 |
| 106 | nonanal | 17.542 | 124-19-6 | 1.212 | 0.897 | 0.819 |
| 107 | benzaldehyde | 20.749 | 100-52-7 | 0.85 | 0.246 | 0.303 |
| 108 | 2-nonenal | 21.186 | 18829-56-6 | 0.547 | 0.602 | 0.237 |
| 109 | propionaldehyde | 2.409 | 123-38-6 | 3.201 | 3.699 | 9.158 |
| 110 | trans-2-heptenal | 15.455 | 18829-55-5 | 0.305 | 0.794 | 0.304 |
| 111 | 2-methyl-2-butenal | 8.176 | 1115-11-3 | 0.088 | 0.057 | 1.944 |
| 112 | 5-hydroxymethyl-2-furfuraldehyde | 40.367 | 67-47-0 | 0.153 | 5.05×10^-6^ | 5.05×10^-6^ |
| 113 | 2-thiophenecarboxaldehyde | 10.193 | 98-3-3 | 0.039 | 5.05×10^-6^ | 5.05×10^-6^ |
| 114 | (Z)-2-heptenal | 15.456 | 57266-86-1 | 0.175 | 0.252 | 5.05×10^-6^ |
| 115 | 2,2-dimethyl- hexanal | 19.150 | 996-12-3 | 0.038 | 5.05×10^-6^ | 0.007 |
| 116 | methyl glyoxal | 28.472 | 78-98-8 | 0.138 | 0.101 | 5.05×10^-6^ |
| 117 | trans-2-hexenal | 12.218 | 6728-26-3 | 5.05×10^-6^ | 2.848 | 5.05×10^-6^ |
| 118 | 2,4-heptadienal | 19.350 | 4313-3-5 | 5.05×10^-6^ | 0.178 | 5.05×10^-6^ |
| 119 | 2,4-decadienal | 27.546 | 2363-88-4 | 0.001 | 0.025 | 0.027 |
| 120 | glyceraldehyde | 13.709 | 56-82-6 | 0.056 | 0.067 | 0.051 |
| 121 | (2E)-2-octenal | 18.481 | 2548-87-0 | 0.766 | 2.128 | 3.205 |
| 122 | 2-hexenal | 12.218 | 505-57-7 | 5.05×10^-6^ | 0.464 | 5.05×10^-6^ |
| 123 | crotonaldehyde | 6.527 | 4170-30-3 | 5.05×10^-6^ | 5.05×10^-6^ | 0.222 |
| 124 | trans-2-pentenal | 9.358 | 1576-87-0 | 0.341 | 5.05×10^-6^ | 1.144 |
| 125 | senecialdehyde | 9.358 | 107-86-8 | 5.05×10^-6^ | 5.05×10^-6^ | 0.291 |
| 126 | (E)-crotonaldehyde | 6.520 | 123-73-9 | 0.076 | 0.025 | 0.209 |
| 127 | 3-hexen-2-one | 12.183 | 763-93-9 | 5.05×10^-6^ | 5.05×10^-6^ | 0.018 |
| 128 | decaldehyde | 20.297 | 112-31-2 | 0.119 | 0.042 | 0.064 |
| 129 | corey aldehyde benzoate | 24.291 | 39746-01-5 | 5.05×10^-6^ | 5.05×10^-6^ | 0.358 |
| 130 | 2-Ethylhexanal ethylene glycol acetal | 21.853 | 0-00-0 | 1.161 | 1.484 | 0.837 |
|  | **Hydrocarbons (51)** |  |  |  |  |  |
| 131 | n-tridecane | 15.103 | 629-50-5 | 2.783 | 2.378 | 2.221 |
| 132 | 1,3-dichlorobenzene | 19.826 | 541-73-1 | 28.037 | 0 | 5.05×10^-6^ |
| 133 | propylene | 1.735 | 115-07-1 | 1.048 | 0.412 | 0.841 |
| 134 | iso-octane | 1.970 | 540-84-1 | 0.132 | 5.05×10^-6^ | 5.05×10^-6^ |
| 135 | n-decane | 5.490 | 124-18-5 | 0.057 | 5.05×10^-6^ | 5.05×10^-6^ |
| 136 | chloroform | 6.005 | 67-66-3 | 2.971 | 2.004 | 1.94 |
| 137 | n-undecane | 8.315 | 1120-21-4 | 0.469 | 0.41 | 0.51 |
| 138 | 4-xylene | 9.332 | 106-42-3 | 0.041 | 0.039 | 0.03 |
| 139 | 3-xylene | 9.502 | 108-38-3 | 0.559 | 0.423 | 0.254 |
| 140 | n-dodecane | 11.775 | 112-40-3 | 1.279 | 0.617 | 0.554 |
| 141 | n-tetradecane | 17.816 | 629-59-4 | 0.048 | 5.05×10^-6^ | 0 |
| 142 | 1,2-dichlorobenzene | 19.830 | 95-50-1 | 53.493 | 51.487 | 44.635 |
| 143 | 2,7,10-trimethyl-dodecane | 22.844 | 74645-98-0 | 0.04 | 5.05×10^-6^ | 5.05×10^-6^ |
| 144 | benzene | 4.013 | 71-43-2 | 0.591 | 0.301 | 5.05×10^-6^ |
| 145 | n-pentadecane | 20.384 | 629-62-9 | 0.068 | 5.05×10^-6^ | 5.05×10^-6^ |
| 146 | 3,3-dimethylhexane | 6.738 | 563-16-6 | 0.065 | 0.049 | 0.075 |
| 147 | 4,7-dimethylundecane | 6.668 | 17301-32-5 | 0.144 | 0.032 | 0.052 |
| 148 | 2-xylene | 9.489 | 95-47-6 | 0.012 | 5.05×10^-6^ | 0.122 |
| 149 | n-hexadecane | 20.393 | 544-76-3 | 0.496 | 0.148 | 0.049 |
| 150 | 3-ethylhexane | 2.939 | 619-99-8 | 0.592 | 0.504 | 0.258 |
| 151 | dichloromethane | 3.826 | 1975-9-2 | 0.872 | 0.194 | 0.184 |
| 152 | 3-methoxypentane | 22.978 | 36839-67-5 | 0.246 | 0.444 | 0.621 |
| 153 | 2,3,6-trimethyl-decane | 18.031 | 62238-12-4 | 0 | 5.05×10^-6^ | 0.007 |
| 154 | farnesane | 13.478 | 3891-98-3 | 0.625 | 5.05×10^-6^ | 0.05 |
| 155 | 4-methyloctane | 2.926 | 2216-34-4 | 0.209 | 5.05×10^-6^ | 0.084 |
| 156 | 2,4,6-trimethyl-decane | 13.476 | 62108-27-4 | 5.05×10^-6^ | 0.35 | 0.016 |
| 157 | (2Z,4Z)-2,4-hexadiene | 2.500 | 6108-61-8 | 5.05×10^-6^ | 0.012± | 0.052± |
| 158 | (1-methylbutyl) oxirane | 16.638 | 53229-39-3 | 5.05×10^-6^ | 0.064± | 5.05×10^-6^ |
| 159 | 2,4-hexadiene | 2.500 | 592-46-1 | 0.015 | 0.015 | 5.05×10^-6^ |
| 160 | 3,5-dimethyloctane | 20.275 | 15869-93-9 | 5.05×10^-6^ | 0.022 | 5.05×10^-6^ |
| 161 | (E)-1-Methyl-3-(prop-1-en-1-yl) trisulfane | 22.731 | 23838-25-7 | 0.136 | 1.551 | 2.103 |
| 162 | 3-methyl-1,4-pentadiene | 1.953 | 1115-08-8 | 5.05×10^-6^ | 0.187 | 0.005 |
| 163 | (2E,4E)-2,4-hexadiene | 2.513 | 5194-51-4 | 5.05×10^-6^ | 0.013 | 0.12 |
| 164 | 4,6-dimethyldodecane | 13.576 | 61141-72-8 | 5.05×10^-6^ | 0.137 | 0.148 |
| 165 | 2-hexyl-1,3-dioxolane | 28.472 | 1708-34-5 | 5.05×10^-6^ | 0.026 | 0.332 |
| 166 | 2,4-dimethyl-decane | 6.607 | 2801-84-5 | 5.05×10^-6^ | 0.014 | 0.027 |
| 167 | (Z)-1-Allyl-2-(prop-1-en-1-yl) disulfane | 19.319 | 122156-03-0 | 0.08 | 1.372 | 7.734 |
| 168 | pentyl-cyclopropane | 21.909 | 2511-91-3 | 5.05×10^-6^ | 5.05×10^-6^ | 0.117 |
| 169 | 2,3,5-trimethyl-decane | 22.848 | 62238-11-3 | 5.05×10^-6^ | 5.05×10^-6^ | 0.046 |
| 170 | butylated hydroxytoluene | 29.680 | 128-37-0 | 5.05×10^-6^ | 5.05×10^-6^ | 0.32 |
| 171 | 3-methylcyclopentene | 1.948 | 1120-62-3 | 0.366 | 0.081 | 0.146 |
| 172 | 1-methylcyclopentene | 2.504 | 693-89-0 | 5.05×10^-6^ | 5.05×10^-6^ | 0.052 |
| 173 | trans-4,6-diethyl-1,2,3,5-tetrathiane | 35.000 | 137363-93-0 | 0.019 | 5.05×10^-6^ | 0.073 |
| 174 | 1-((E)-Prop-1-en-1-yl)-2-((Z)-prop-1-en-1-yl) disulfane | 19.254 | 121609-82-3 | 0.147 | 0.179 | 0.173 |
| 175 | 2-methyl-1-heptene | 16.638 | 15870-10-7 | 5.05×10^-6^ | 5.05×10^-6^ | 0.038 |
| 176 | 3-ethyl-3-methylheptane | 6.590 | 17302-01-1 | 5.05×10^-6^ | 0.016 | 0.031 |
| 177 | cis-4,6-diethyl-1,2,3,5-tetrathiane | 35.000 | 137363-91-8 | 0.035 | 5.05×10^-6^ | 0.297 |
| 178 | 2-(3-bromo-5,5,5-trichloro-2,2-dimethylpentyl)-1,3-dioxolane | 28.476 | 0-00-0 | 0.068 | 0.073 | 5.05×10^-6^ |
| 179 | 3-methoxy-4-methylheptane | 22.974 | 0-00-0 | 0.111 | 0 | 5.05×10^-6^ |
| 180 | cis-4,5-epoxy-(E)-2-decenal | 31.536 | 0-00-0 | 5.05×10^-6^ | 0.134 | 5.05×10^-6^ |
| 181 | 2,3,3-trimethyl-1-hexene | 9.354 | 0-00-0 | 5.05×10^-6^ | 0.016 | 5.05×10^-6^ |
|  | **nitrogen and its derivatives (39)** |  |  |  |  |  |
| 182 | trimethyloxamine | 2.183 | 1184-78-7 | 0.164 | 5.05×10^-6^ | 5.05×10^-6^ |
| 183 | tetrabutylammonium | 2.396 | 10549-76-5 | 0.462 | 0.641 | 5.05×10^-6^ |
| 184 | N-(4-Piperidinyl) cyclohexanecarboxamide | 9.360 | 78555-36-9 | 0.511 | 0.32 | 5.05×10^-6^ |
| 185 | guanidine | 2.572 | 113-00-8 | 5.05×10^-6^ | 0.475 | 0.304 |
| 186 | amphetamine-D5 | 19.254 | 136765-27-0 | 0.037 | 5.05×10^-6^ | 5.05×10^-6^ |
| 187 | carbamoylcholine | 28.337 | 462-58-8 | 0.024 | 5.05×10^-6^ | 5.05×10^-6^ |
| 188 | aminoimidazole carboxamide | 19.793 | 360-97-4 | 39.204 | 21.426 | 24.418 |
| 189 | moclobemide | 6.448 | 71320-77-9 | 0.115 | 0.002 | 0.005 |
| 190 | acetazolamide | 16.933 | 59-66-5 | 0.442 | 5.05×10^-6^ | 5.05×10^-6^ |
| 191 | propoxycarbazone | 12.659 | 145026-81-9 | 0.153 | 5.05×10^-6^ | 0.299 |
| 192 | lisofylline | 19.823 | 100324-81-0 | 2.955 | 6.595 | 5.05×10^-6^ |
| 193 | entacapone | 20.771 | 130929-57-6 | 0.442 | 0.412 | 0.668 |
| 194 | hydroxyurea | 24.302 | 127-07-1 | 0.403 | 0.426 | 10.682 |
| 195 | oxabetrinil | 40.502 | 74782-23-3 | 4.336 | 5.05×10^-6^ | 5.05×10^-6^ |
| 196 | vildagliptin | 14.825 | 274901-16-5 | 0.074 | 0.007 | 5.05×10^-6^ |
| 197 | sulfabenzamide | 18.641 | 127-71-9 | 16.147 | 5.05×10^-6^ | 5.05×10^-6^ |
| 198 | alpha-cyano-(3,4-dihydroxy)-n-benzylcinnamide | 18.698 | 133550-30-8 | 15.009 | 9.357 | 5.05×10^-6^ |
| 199 | n,n-dimethylformamide | 19.828 | 68-12-2 | 5.05×10^-6^ | 5.05×10^-6^ | 10.585 |
| 200 | (E)-3-(p-toluenesulfonyl) acrylonitrile | 29.706 | 19542-67-7 | 0.207 | 0.371 | 5.05×10^-6^ |
| 201 | 3-((2-ethylhexyl) oxy) propylamine | 2.403 | 5397-31-9 | 0.94 | 0.561 | 0.002 |
| 202 | asparagine | 24.335 | 70-47-3 | 0.657 | 5.05×10^-6^ | 5.05×10^-6^ |
| 203 | mexiletine | 26.047 | 31828-71-4 | 5.663 | 0.001 | 5.05×10^-6^ |
| 204 | proximphan | 29.759 | 2828-42-4 | 0.258 | 5.05×10^-6^ | 5.05×10^-6^ |
| 205 | diflufenzopyr | 30.528 | 109293-97-2 | 8.38 | 5.05×10^-6^ | 5.05×10^-6^ |
| 206 | ammonium acetate | 19.193 | 631-61-8 | 0.046 | 0.115 | 5.05×10^-6^ |
| 207 | tripropylamine | 1.744 | 102-69-2 | 5.05×10^-6^ | 1.775 | 0.887 |
| 208 | n-acetyl-m-aminophenol | 17.068 | 621-42-1 | 5.05×10^-6^ | 30.884 | 5.145 |
| 209 | (1R,5S)-9-Methyl-9-azabicyclo [3.3.1] nonan-3-amine | 1.955 | 141650-55-7 | 0.373 | 0.509 | 5.05×10^-6^ |
| 210 | n~4- [2-(4- phenoxyphenyl) ethyl] quinazoline-4,6-diamine | 30.589 | 545380-34-5 | 0.486 | 0.519 | 5.05×10^-6^ |
| 211 | acetonitrile | 3.543 | 75-5-8 | 0.038 | 0.333 | 0.071 |
| 212 | dodine | 6.746 | 112-65-2 | 5.05×10^-6^ | 0.006 | 5.05×10^-6^ |
| 213 | n,n-bis(dichloroacetyl)-1,8-octamethylenediamine | 3.513 | 1477-57-2 | 5.05×10^-6^ | 5.05×10^-6^ | 1.352 |
| 214 | naratriptan n-oxide | 24.404 | 1159977-52-2 | 5.05×10^-6^ | 5.05×10^-6^ | 7.43 |
| 215 | lacosamide | 30.115 | 175481-36-4 | 5.05×10^-6^ | 5.05×10^-6^ | 0.008 |
| 216 | flutamide | 26.744 | 13311-84-7 | 0.297 | 5.05×10^-6^ | 0.54 |
| 217 | ammonium carbamate | 1.713 | 1111-78-0 | 2.28 | 2.632 | 2.654 |
| 218 | 2-nitrobenzaldehyde semicarbazone | 2.444 | 16004-43-6 | 5.05×10^-6^ | 5.05×10^-6^ | 0.339 |
| 219 | ammeline | 18.520 | 645-92-1 | 5.05×10^-6^ | 5.05×10^-6^ | 1.016 |
| 220 | 2-bromo-4,5-methylenedioxymethamphetamine | 4.021 | 0-00-0 | 0.954 | 1.095 | 3.77 |
|  | **organic acids and its derivatives (43)** |  |  |  |  |  |
| 221 | 9-hydroxy-10,12,15-octadecatrienoic acid | 24.300 | 89886-42-0 | 1.103 | 0.238 | 0.504 |
| 222 | decyl trifluoroacetate | 30.945 | 333-88-0 | 0.037 | 5.05×10^-6^ | 5.05×10^-6^ |
| 223 | 2-[(2-bromophenyl) methoxy]-Benzoic acid | 3.491 | 743456-83-9 | 3.364 | 0.357 | 0.237 |
| 224 | 3-mercaptopyruvic acid | 6.442 | 2464-23-5 | 0.035 | 5.05×10^-6^ | 0.002± |
| 225 | o-methylcinnamic acid | 28.116 | 2373-76-4 | 0.286 | 5.05×10^-6^ | 5.05×10^-6^ |
| 226 | mandelic acid | 2.005 | 90-64-2 | 0.229 | 5.05×10^-6^ | 5.05×10^-6^ |
| 227 | hydroxyacetic acid | 24.200 | 79-14-1 | 0.108 | 0.01 | 5.05×10^-6^ |
| 228 | metoprolol acid | 24.409 | 56392-14-4 | 8.166 | 5.05×10^-6^ | 2.129 |
| 229 | 3,6,9-trioxaundecanedioic acid | 2.018 | 13887-98-4 | 0.616 | 0.918 | 5.05×10^-6^ |
| 230 | alpha-aminoadipic acid | 2.794 | 542-32-5 | 0.276 | 0.09 | 0.007 |
| 231 | n-(2-acetamido) iminodiacetic acid | 16.955 | 26239-55-4 | 0.362 | 25.764 | 113.015 |
| 232 | (2-(2-methoxyethoxy) ethoxy) acetic acid | 18.528 | 16024-58-1 | 3.464 | 5.05×10^-6^ | 5.05×10^-6^ |
| 233 | myristic acid | 43.072 | 544-63-8 | 0.345 | 0.125 | 0.124 |
| 234 | levulinic acid | 15.554 | 123-76-2 | 0.275 | 0.157 | 0.086 |
| 235 | n-butyric acid | 17.025 | 107-92-6 | 8.62 | 5.05×10^-6^ | 4.67 |
| 236 | caftaric acid | 28.607 | 67879-58-7 | 0.071 | 0.235 | 0.022 |
| 237 | 2,3-diaminopropionic acid | 24.333 | 515-94-6 | 0.393 | 0.809 | 5.05×10^-6^ |
| 238 | acetic anhydride | 24.391 | 108-24-7 | 5.05×10^-6^ | 2.643 | 0.994 |
| 239 | 2-amino-3-phosphonopropionic acid | 17.307 | 5652-28-8 | 5.05×10^-6^ | 46.466 | 5.05×10^-6^ |
| 240 | hippuric acid | 25.964 | 495-69-2 | 5.05×10^-6^ | 1.799 | 5.05×10^-6^ |
| 241 | guanidinopropionic acid | 29.628 | 353-09-3 | 5.05×10^-6^ | 0.084 | 0.03 |
| 242 | 10-camphorsulfonic acid | 24.339 | 5872-8-2 | 5.05×10^-6^ | 0.289 | 5.05×10^-6^ |
| 243 | 2-(2-butoxyethoxy)-acetic acid | 2.018 | 82941-26-2 | 0.478 | 0.632 | 0.642 |
| 244 | aminomethanesulfonic acid | 3.635 | 13881-91-9 | 1.96×10^-04^ | 0.215 | 5.05×10^-6^ |
| 245 | allantoic acid | 24.196 | 99-16-1 | 5.05×10^-6^ | 0.051 | 5.05×10^-6^ |
| 246 | 3-methylflavone-8-carboxylic acid | 5.834 | 3468-1-7 | 0.5 | 5.05×10^-6^ | 0.257 |
| 247 | hexanoic acid | 12.548 | 142-62-1 | 5.05×10^-6^ | 5.05×10^-6^ | 1.983 |
| 248 | bes | 35.261 | 10191-18-1 | 5.05×10^-6^ | 5.05×10^-6^ | 0.195 |
| 249 | acetic acid--hex-4-en-1-ol | 2.643 | 72237-36-6 | 0.064 | 0.013 | 0.054 |
| 250 | 5-oxo-d-gluconic acid | 12.318 | 5287-64-9 | 5.05×10^-6^ | 5.05×10^-6^ | 0.116 |
| 251 | vigabatrin | 19.771 | 60643-86-9 | 5.05×10^-6^ | 5.05×10^-6^ | 5.806 |
| 252 | loxoprofen | 27.181 | 68767-14-6 | 5.05×10^-6^ | 5.05×10^-6^ | 3.203 |
| 253 | 3-chloro-4-hydroxy-5-methoxybenzoic acid | 18.663 | 62936-23-6 | 5.05×10^-6^ | 5.05×10^-6^ | 5.923 |
| 254 | trimellitic anhydride | 19.813 | 552-30-7 | 5.05×10^-6^ | 5.05×10^-6^ | 11.778 |
| 255 | nonanoic acid | 34.963 | 112-05-0 | 0.206 | 0.254 | 0.114 |
| 256 | rhein | 40.541 | 478-43-3 | 5.05×10^-6^ | 1.177 | 1.439 |
| 257 | dl-pantothenic acid | 40.522 | 599-54-2 | 5.05×10^-6^ | 5.05×10^-6^ | 1.996 |
| 258 | fusaric acid | 26.703 | 536-69-6 | 5.05×10^-6^ | 5.05×10^-6^ | 2.241 |
| 259 | guanidinosuccinic acid | 17.103 | 6133-30-8 | 18.19 | 5.05×10^-6^ | 14.941 |
| 260 | acetic acid | 19.191 | 64-19-7 | 0.006 | 0.024 | 0.069 |
| 261 | propionic acid | 21.488 | 79-9-4 | 5.05×10^-6^ | 0.102 | 5.05×10^-6^ |
| 262 | isovaleric acid | 24.684 | 503-74-2 | 0.075 | 0.003 | 5.05×10^-6^ |
| 263 | succinic acid | 2.005 | 110-15-6 | 0.184 | 5.05×10^-6^ | 5.05×10^-6^ |
|  | **esters (60)** |  |  |  |  |  |
| 264 | 1,3-epoxypropane | 2.396 | 503-30-0 | 1.803 | 5.05×10^-6^ | 5.05×10^-6^ |
| 265 | methyl acetate | 2.657 | 79-20-9 | 0.188 | 0.135 | 0.369 |
| 266 | ethyl acetate | 3.226 | 141-78-6 | 0.091 | 0.033 | 0.018 |
| 267 | 2,2,4-trimethyl-1,3-pentanediol diisobutyrate | 29.028 | 6846-50-0 | 0.49 | 0.519 | 0.274 |
| 268 | methyl palmitate | 35.621 | 112-39-0 | 3.264 | 2.377 | 2.768 |
| 269 | pentanoic acid, 5-hydroxy-, 2,4-di-t-butylphenyl esters | 37.242 | 166273-38-7 | 0.356 | 0.367 | 0.074 |
| 270 | 11,14-eicosadienoic acid, methyl ester | 40.363 | 2463-2-7 | 0.032 | 5.05×10^-6^ | 5.05×10^-6^ |
| 271 | dibutyl phthalate | 42.901 | 84-74-2 | 1.194 | 0.985 | 0.858 |
| 272 | s-propyl propanethioate | 13.041 | 2432-43-1 | 0.656 | 0.565 | 0.234 |
| 273 | (Z)-prop-1-en-1-yl propanedithioate | 21.507 | 67230-81-3 | 0.994 | 1.124 | 1.474 |
| 274 | octyl formate | 21.914 | 112-32-3 | 0.101 | 0.092 | 5.05×10^-6^ |
| 275 | methyl myristate | 31.699 | 124-10-7 | 3.052 | 1.895 | 1.316 |
| 276 | methyl oleate | 39.589 | 112-62-9 | 0.357 | 5.05×10^-6^ | 5.05×10^-6^ |
| 277 | dimethyl diethylmalonate | 41.371 | 27132-23-6 | 16.736 | 14.329 | 13.844 |
| 278 | methyl acetoacetate | 16.996 | 105-45-3 | 7.768 | 5.926 | 5.05×10^-6^ |
| 279 | 2-hydroxyethyl acrylate | 20.258 | 818-61-1 | 0.027 | 5.05×10^-6^ | 5.05×10^-6^ |
| 280 | cyclopentolate | 30.510 | 512-15-2 | 4.319 | 5.05×10^-6^ | 5.05×10^-6^ |
| 281 | tert-butyl (5-chloro-2,2-dioxospiro[indole-3,3-pyrrolidin]-1(2h)-yl) acetate | 17.359 | 916048-02-7 | 36.775 | 5.05×10^-6^ | 5.05×10^-6^ |
| 282 | propyl carbonotrithioate | 3.369 | 68060-07-1 | 0.049 | 0.014 | 0.256 |
| 283 | dimetilan | 16.916 | 644-64-4 | 0.246 | 5.05×10^-6^ | 5.05×10^-6^ |
| 284 | prallethrin | 25.126 | 23031-36-9 | 0.067 | 5.05×10^-6^ | 5.05×10^-6^ |
| 285 | 3,6-dimethyl-4-methoxycoumarin | 29.719 | 720675-55-8 | 0.242 | 5.05×10^-6^ | 0.005 |
| 286 | hexanethioic acid, S-heptyl ester | 20.860 | 2432-80-6 | 5.05×10^-6^ | 0.027 | 0.003 |
| 287 | benzoic acid, 4-hydroxy-, 1-methylethyl ester | 24.204 | 4191-73-5 | 5.05×10^-6^ | 0.04 | 5.05×10^-6^ |
| 288 | propamocarb | 30.541 | 24579-73-5 | 8.986 | 9.856 | 5.05×10^-6^ |
| 289 | hexyl formate | 16.638 | 629-33-4 | 5.05×10^-6^ | 0.16 | 5.05×10^-6^ |
| 290 | sec-butyl thiohexanoate | 20.856 | 2432-79-3 | 5.05×10^-6^ | 0.073 | 5.05×10^-6^ |
| 291 | N-benzyl-L-methionine methyl ester | 28.359 | 452282-27-8 | 5.05×10^-6^ | 0.772 | 5.05×10^-6^ |
| 292 | fyrol pcf | 28.398 | 6145-73-9 | 0.325 | 0.787 | 5.05×10^-6^ |
| 293 | methyl propionate | 3.482 | 554-12-1 | 4.408 | 4.544 | 3.448 |
| 294 | propanethioic acid, S-(1-methylethyl) ester | 13.035 | 2432-47-5 | 5.05×10^-6^ | 0.085 | 5.05×10^-6^ |
| 295 | ethyl pyruvate | 10.484 | 617-35-6 | 0.033 | 0.268 | 5.05×10^-6^ |
| 296 | dicamba-methyl | 19.806 | 6597-78-0 | 5.05×10^-6^ | 11.361 | 4.518 |
| 297 | 3-(hexadecyloxy)-2-hydroxypropyl dihydrogen phosphate | 28.398 | 52603-03-9 | 5.05×10^-6^ | 0.561 | 5.05×10^-6^ |
| 298 | propyl thioacetate | 10.914 | 2307-10-0 | 5.05×10^-6^ | 0.166 | 0.069 |
| 299 | butanoic acid, 4-(2,4-dichlorophenoxy)-, methyl ester | 2.413 | 18625-12-2 | 5.05×10^-6^ | 0.686 | 5.05×10^-6^ |
| 300 | decyl chloroformate | 16.469 | 55488-51-2 | 5.05×10^-6^ | 0.168 | 5.05×10^-6^ |
| 301 | methyl 3-[methyl(nitroso)amino] propanoate | 26.669 | 383417-47-8 | 5.05×10^-6^ | 5.05×10^-6^ | 1.536 |
| 302 | sulfallate | 28.337 | 95-6-7 | 5.05×10^-6^ | 5.05×10^-6^ | 0.015 |
| 303 | propanoic acid, 2-hydroxy-2-methyl-, methyl ester | 16.018 | 2110-78-3 | 5.05×10^-6^ | 0.082 | 0.237 |
| 304 | tri-(2-chloroisopropyl) phosphate | 30.975 | 13674-84-5 | 5.05×10^-6^ | 0.005 | 0.012 |
| 305 | N-benzyl-D-methionine methyl ester | 1.731 | 1273387-38-4 | 5.05×10^-6^ | 5.05×10^-6^ | 0.367 |
| 306 | methyl methoxyacetate | 4.056 | 6290-49-9 | 5.05×10^-6^ | 5.05×10^-6^ | 0.21 |
| 307 | clasto-lactacystin î²-lactone | 10.253 | 154226-60-5 | 5.05×10^-6^ | 5.05×10^-6^ | 0.219 |
| 308 | 3-hexenylacetate | 2.648 | 3681-71-8 | 0.004 | 0.081 | 0.099 |
| 309 | n-(beta-oxooctan-1-oyl) homoserine lactone | 13.048 | 147795-39-9 | 0.01 | 5.05×10^-6^ | 0.021 |
| 310 | 4,7-dimethyl-3-phenylcoumarin | 22.757 | 218932-55-9 | 5.05×10^-6^ | 5.05×10^-6^ | 0.104 |
| 311 | ethyl tyrosine ester | 29.702 | 949-67-7 | 5.05×10^-6^ | 5.05×10^-6^ | 0.014 |
| 312 | 5-hydroxy-dl-lysine | 8.307 | 6000-8-4 | 5.05×10^-6^ | 5.05×10^-6^ | 0.025 |
| 313 | promecarb | 29.698 | 2631-37-0 | 5.05×10^-6^ | 5.05×10^-6^ | 0.027 |
| 314 | methyl octadeca-9,12-dienoate | 40.363 | 2462-85-3 | 5.05×10^-6^ | 5.05×10^-6^ | 0.059 |
| 315 | n-heptanoyl-dl-homoserine lactone | 24.335 | 106983-26-0 | 5.05×10^-6^ | 5.05×10^-6^ | 0.371 |
| 316 | S-propyl propane-1-sulfonothioate | 33.579 | 0-00-0 | 1.1 | 5.05×10^-6^ | 5.05×10^-6^ |
| 317 | phthalic acid, hept-4-yl isobutyl ester | 41.089 | 0-00-0 | 0.454 | 0.243 | 0.189 |
| 318 | (E)-hex-3-enyl (E)-2-methylbut-2-enoate | 22.918 | 0-00-0 | 0.007 | 0.004 | 5.05×10^-6^ |
| 319 | propyl pyruvate | 24.439 | 0-00-0 | 5.69 | 5.05×10^-6^ | 5.05×10^-6^ |
| 320 | phthalic acid, isobutyl 4-octyl ester | 41.089 | 0-00-0 | 5.05×10^-6^ | 0.088 | 0.025 |
| 321 | phthalic acid, isobutyl 2-pentyl ester | 41.089 | 0-00-0 | 5.05×10^-6^ | 0.12 | 5.05×10^-6^ |
| 322 | phthalic acid, cyclohexyl isohexyl ester | 41.084 | 0-00-0 | 5.05×10^-6^ | 5.05×10^-6^ | 0.027 |
| 323 | sulfurous acid, 2-ethylhexyl isohexyl ester | 20.384 | 0-00-0 | 5.05×10^-6^ | 5.05×10^-6^ | 0.18 |
|  | **Heterocycles (27)** |  |  |  |  |  |
| 324 | 2,5-dimethylpyrazine | 15.512 | 123-32-0 | 0.444 | 0.373 | 5.05×10^-6^ |
| 325 | 3,4-dimethylthiophene | 13.200 | 632-15-5 | 5.343 | 6.273 | 12.041 |
| 326 | 2,6-dimethylpyrazine | 15.695 | 108-50-9 | 0.381 | 0.035 | 0.11 |
| 327 | 2,3,5-trimethylpyrazine | 17.887 | 14667-55-1 | 0.157 | 5.05×10^-6^ | 5.05×10^-6^ |
| 328 | lansoprazole | 3.526 | 103577-45-3 | 0.564 | 5.05×10^-6^ | 0.647 |
| 329 | 2-amylfuran | 12.661 | 3777-69-3 | 3.653 | 2.961 | 2.626 |
| 330 | l-hydroorotic acid | 17.094 | 5988-19-2 | 14.114 | 5.05×10^-6^ | 9.033 |
| 331 | 1-phenylpyrrolidine | 18.524 | 4096-21-3 | 3.405 | 5.05×10^-6^ | 5.05×10^-6^ |
| 332 | 3-[(naphthalen-1-yl) methyl]-1-pentyl-1h-indole | 35.978 | 619294-35-8 | 0.216 | 5.05×10^-6^ | 5.05×10^-6^ |
| 333 | 2,5-dimethylthiophene | 11.240 | 638-02-8 | 5.05×10^-6^ | 0.074 | 0.212± |
| 334 | 1-methyl-3-isobutylxanthine | 19.793 | 28822-58-4 | 12.641 | 5.05×10^-6^ | 5.05×10^-6^ |
| 335 | 5-hydroxythiabendazole | 19.815 | 948-71-0 | 15.905 | 13.353 | 10.038 |
| 336 | guanazole | 28.398 | 1455-77-2 | 1.202 | 5.05×10^-6^ | 5.05×10^-6^ |
| 337 | 2-methoxyfuran | 12.185 | 25414-22-6 | 0.083 | 5.05×10^-6^ | 0.088 |
| 338 | chlorpromazine | 37.247 | 50-53-3 | 0.067 | 0.039 | 5.05×10^-6^ |
| 339 | lofexidine | 10.475 | 31036-80-3 | 5.05×10^-6^ | 0.493 | 5.05×10^-6^ |
| 340 | methimazole | 25.999 | 60-56-0 | 5.05×10^-6^ | 2.353 | 5.05×10^-6^ |
| 341 | 4-methyl-2,3-dihydrofuran | 8.167 | 34314-83-5 | 0.179 | 0.05 | 0.039 |
| 342 | 2,4-dimethylthiophene | 11.153 | 638-00-6 | 5.05×10^-6^ | 5.05×10^-6^ | 0.863 |
| 343 | 3-iodo-1h-pyrazole | 10.232 | 4522-35-4 | 5.05×10^-6^ | 5.05×10^-6^ | 0.09 |
| 344 | 3-amino-5-(4-morpholinomethyl) oxazolidin-2-one | 2.144 | 43056-63-9 | 5.05×10^-6^ | 0.921 | 1.954 |
| 345 | 2,4-dimethylfuran | 4.580 | 3710-43-8 | 0.001± | 5.05×10^-6^ | 0.081 |
| 346 | 4-methyl-2-(4-methyl-6-oxo-1-oxahexyl) tetrahydro-2H-pyran | 28.402 | 101153-83-7 | 1.154,27 | 0.626,77 | 1.455,57 |
| 347 | 2,3-dimethylthiophene | 10.202 | 632-16-6 | 0.008 | 5.05×10^-6^ | 0.011 |
| 348 | 10-oxomorphine | 24.396 | 68254-48-8 | 5.05×10^-6^ | 5.05×10^-6^ | 3.255 |
| 349 | 2-Mercapto-3,4-dimethyl-2,3-dihydrothiophene | 25.247 | 0-00-0 | 1.686 | 2.736 | 5.561 |
| 350 | 3,4-dimethylfuran | 4.591 | 0-00-0 | 0.005 | 0.014 | 0.044 |
|  | **Others (40)** |  |  |  |  |  |
| 351 | pinostilbene | 5.955 | 42438-89-1 | 0.036 | 5.05×10^-6^ | 5.05×10^-6^ |
| 352 | 2-amino-4-nitrophenol | 3.552 | 99-57-0 | 5.05×10^-6^ | 0.352 | 5.05×10^-6^ |
| 353 | 3-methyluridine | 25.156 | 6038-59-1 | 0.031 | 5.05×10^-6^ | 5.05×10^-6^ |
| 354 | cytidine | 26.047 | 65-46-3 | 5.05×10^-6^ | 3.925 | 5.05×10^-6^ |
| 355 | TRH | 24.409 | 0-00-0 | 17.18 | 5.05×10^-6^ | 5.05×10^-6^ |
| 356 | tetraethyl-phosphonium | 2.615 | 13983-95-4 | 0.959 | 0.013 | 5.05×10^-6^ |
| 357 | diethyl ether | 1.885 | 60-29-7 | 4.706 | 0.991 | 0.2650.75 |
| 358 | dowanol tpm | 23.378 | 25498-49-1 | 0.104 | 5.05×10^-6^ | 5.05×10^-6^ |
| 359 | 1-methoxy-2-hydroxypropane | 9.663 | 107-98-2 | 0.074 | 0.011 | 0.005 |
| 360 | methyl pentadecanoate | 33.700 | 7132-64-1 | 0.331 | 0.216 | 0.271 |
| 361 | ethylene glycol diethyl ether | 1.892 | 629-14-1 | 5.05×10^-6^ | 1.645 | 1.455 |
| 362 | ligustrazine | 19.760 | 1124-11-4 | 0.263 | 5.05×10^-6^ | 5.05×10^-6^ |
| 363 | dl-glyceraldehyde 3-phosphate | 30.454 | 591-59-3 | 0.012 | 5.05×10^-6^ | 5.05×10^-6^ |
| 364 | n-acetylglucosaminylasparagine | 10.384 | 2776-93-4 | 5.05×10^-6^ | 0.548 | 5.05×10^-6^ |
| 365 | l-fucose | 19.819 | 2438-80-4 | 0.945 | 4.73 | 5.05×10^-6^ |
| 366 | rosin | 29.697 | 85026-55-7 | 5.05×10^-6^ | 5.05×10^-6^ | 0.065 |
| 367 | 3-Galactobiose-CH3 | 24.741 | 0-00-0 | 0.086 | 0.033 | 5.05×10^-6^ |
| 368 | (d)-limonene | 11.453 | 5989-27-5 | 1.16 | 1.282 | 0.6 |
| 369 | gamma-terpinene | 12.974 | 99-85-4 | 0.016 | 0.017 | 5.05×10^-6^ |
| 370 | acetone | 2.561 | 67-64-1 | 0.264 | 0.203 | 0.048 |
| 371 | 3-octanone | 13.426 | 106-68-3 | 0.233 | 5.05×10^-6^ | 5.05×10^-6^ |
| 372 | acetophenone | 23.874 | 98-86-2 | 0.046 | 5.05×10^-6^ | 5.05×10^-6^ |
| 373 | boldione | 13.018 | 897-06-3 | 0.011 | 5.05×10^-6^ | 5.05×10^-6^ |
| 374 | 2-hydroxypentan-3-one | 16.486 | 5704-20-1 | 0.049 | 0.085 | 0.187 |
| 375 | 4,9-dihydro-3-methyl-1h-purine-2,6,8(3h)-trione | 10.610 | 605-99-2 | 0.022 | 5.05×10^-6^ | 5.05×10^-6^ |
| 376 | 6,3-dimethoxy-3-hydroxyflavone | 24.408 | 720676-20-0 | 7.479 | 5.05×10^-6^ | 5.05×10^-6^ |
| 377 | 2-heptanone | 11.144 | 110-43-0 | 0.021 | 5.05×10^-6^ | 5.05×10^-6^ |
| 378 | 1-(2-Nitrobenzylidenamino)-2,4-imidazolidinedione | 2.796 | 1228184-63-1 | 0.674 | 0.017 | 0.019 |
| 379 | pindone | 39.168 | 83-26-1 | 0.138 | 0.11 | 0.1 |
| 380 | 1-methylxanthine | 19.802 | 6136-37-4 | 5.05×10^-6^ | 4.73 | 5.05×10^-6^ |
| 381 | dexrazoxane | 17.077 | 24584-09-6 | 5.05×10^-6^ | 4.996 | 5.05×10^-6^ |
| 382 | efavirenz | 24.411 | 154598-52-4 | 5.05×10^-6^ | 5.05×10^-6^ | 9.317 |
| 383 | 4-methyl-3h-1,2-dithiole-3-thione | 39.411 | 3354-41-4 | 0.04 | 0.093 | 0.731 |
| 384 | 2-amino-4-methylbenzophenone | 18.602 | 4937-62-6 | 5.05×10^-6^ | 5.05×10^-6^ | 3.723 |
| 385 | 4-methyl-1,2-dithiol-3-one | 30.725 | 3620-10-8 | 5.05×10^-6^ | 0.015 | 0.289 |
| 386 | 4-(n-nitrosomethylamino)-1-(3-pyridyl)-1-butanone | 12.713 | 64091-91-4 | 1.279 | 5.05×10^-6^ | 0.47 |
| 387 | 2-undecanone | 22.788 | 112-12-9 | 0.104 | 5.05×10^-6^ | 0.097 |
| 388 | 2-hexyl-5-methyl-3(2h)-furanone | 31.449 | 33922-66-6 | 5.05×10^-6^ | 0.037 | 0.064 |
| 389 | perchloric acid | 28.394 | 7601-90-3 | 0.519 | 5.05×10^-6^ | 5.05×10^-6^ |
| 390 | piperalin | 18.116 | 3478-94-2 | 5.05×10^-6^ | 0.134 | 5.05×10^-6^ |

| Table S2 Performance test of four machine learning algorithms for classification of metabolites | | | | | |
| --- | --- | --- | --- | --- | --- |
| Model | Type | Precision | Recall | F1-score | Accuracy |
| XG Boost | White | 0.67 | 0.75 | 0.71 | 0.75 |
|  | Yellow | 0.62 | 0.62 | 0.62 |  |
|  | Red | 1 | 0.88 | 0.93 |  |
| Random Forest | White | 1 | 1 | 1 | 1 |
|  | Yellow | 1 | 1 | 1 |  |
|  | Red | 1 | 1 | 1 |  |
| Logistic Regression | White | 1 | 0.88 | 0.93 | 0.96 |
|  | Yellow | 0.89 | 1 | 0.94 |  |
|  | Red | 1 | 1 | 1 |  |
| Decision Tree | White | 0.71 | 0.62 | 0.67 | 0.71 |
|  | Yellow | 0.55 | 0.75 | 0.63 |  |
|  | Red | 1 | 0.75 | 0.86 |  |


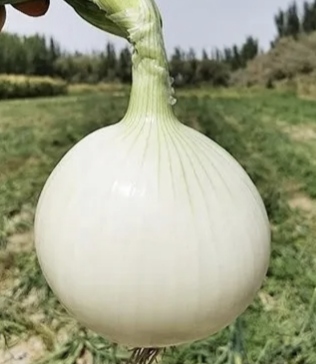

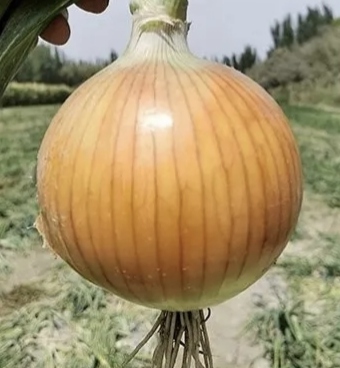

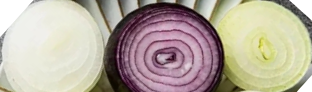

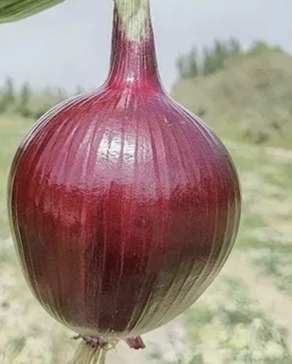


**Fig. S1 Appearance and transverse images of the three colored onion varieties.**

Baibilong (white-colored), Hongyou 1(red-colored), and Jinke 7 (yellow-colored)

**White**

**Red**

**Yellow**















**F**

**E**

**D**

**C**

**B**

**A**

**Fig. S2 Comparison of major nutrients and antioxidant activity of three colored onions. (A) total anthocyanins content (B) total flavonoids content (C) Total phenol content (D) carotenoids (E) DPPH radical scavenging rate (F) ABTS radical scavenging rate. Different lowercase letters in column indicate significant difference(P<0.05).**


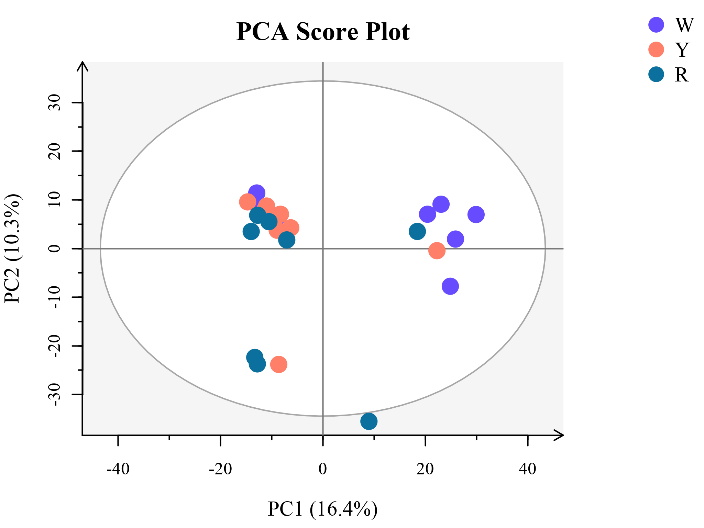

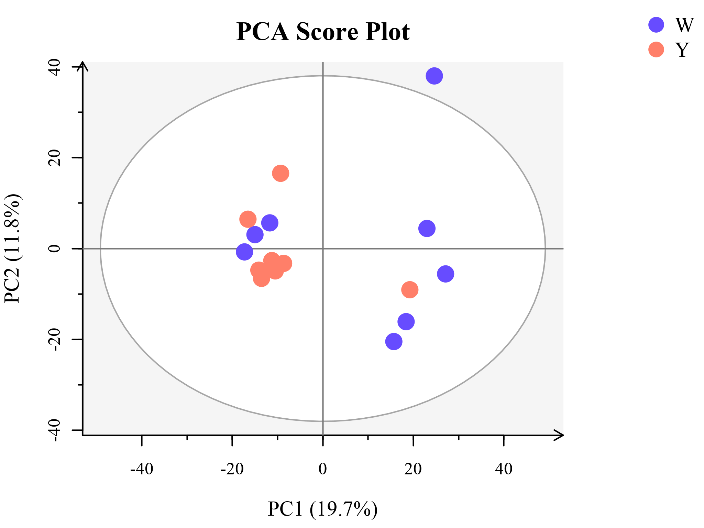

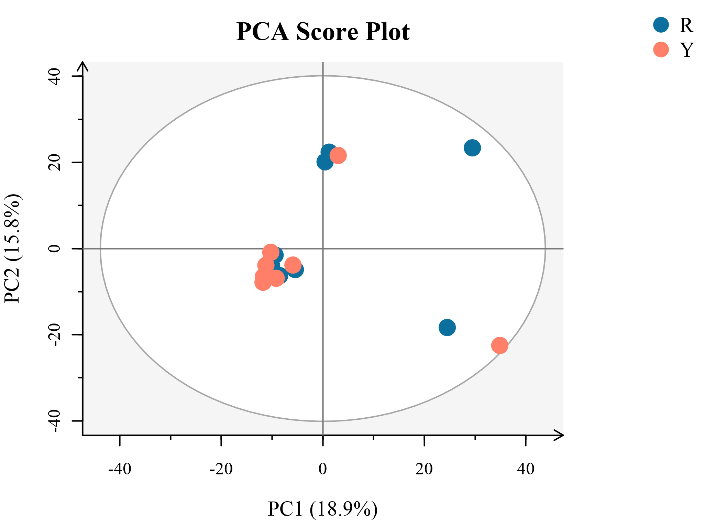

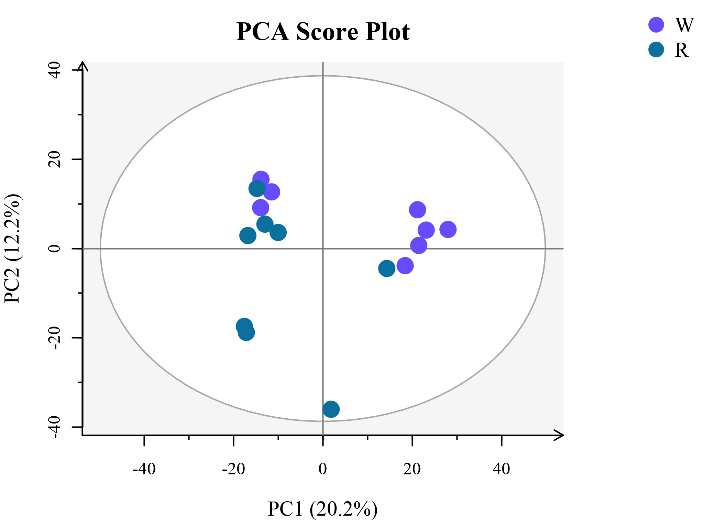


**D**

**C**

**B**

**A**

**Fig. S3** PCA score plots of volatile metabolites in three colored onions. (A) white-colored vs red-colored (B) red-colored vs yellow-colored (C) white-colored vs yellow-colored (D) white-colored vs yellow-colored vs red-colored


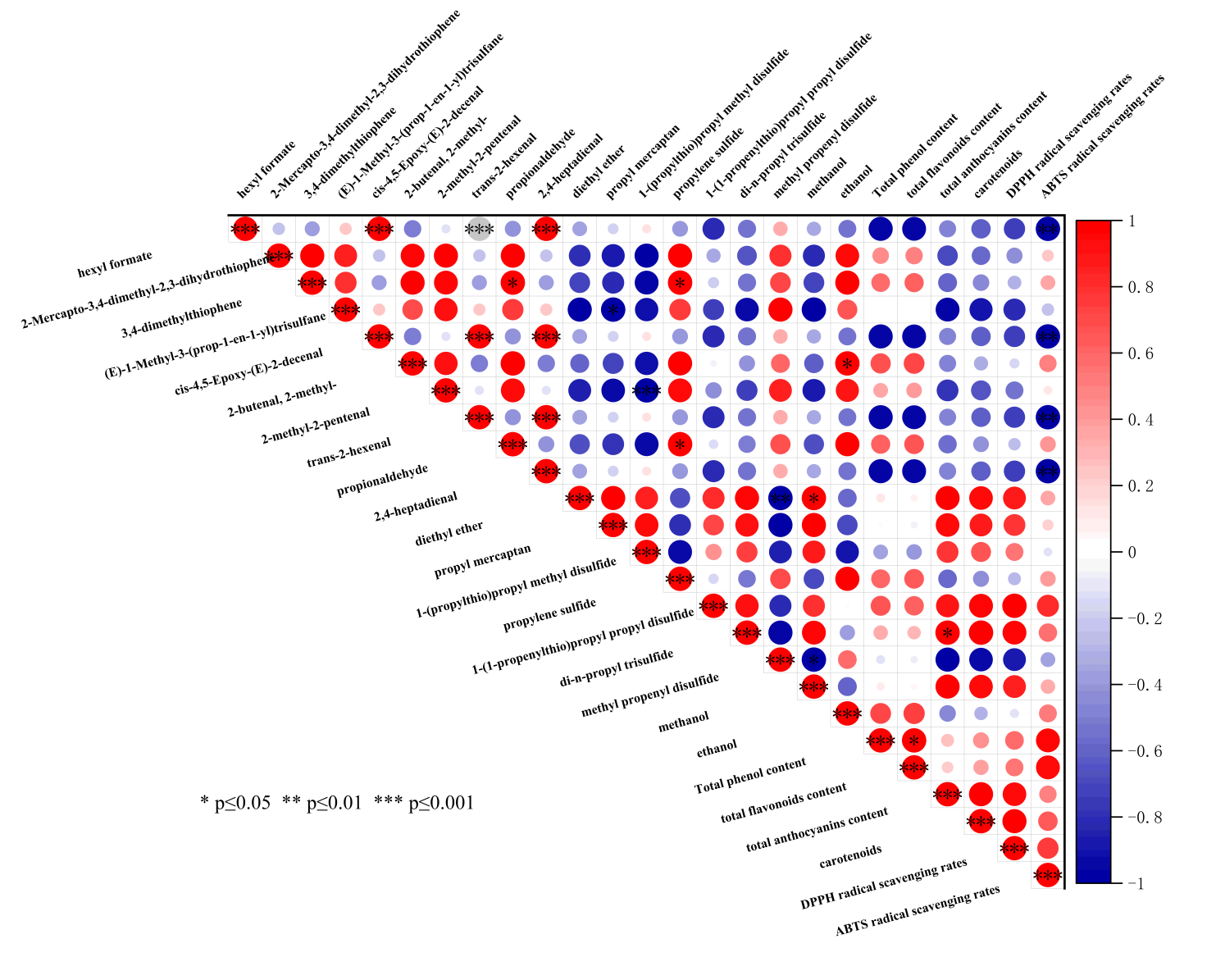


**Fig. S4** Pearson correlation analysis of differential metabolites, major nutrients, and antioxidant activities in colored onions. * Denotes significant difference at 0.05 level. ** denotes significant difference at 0.01 level.


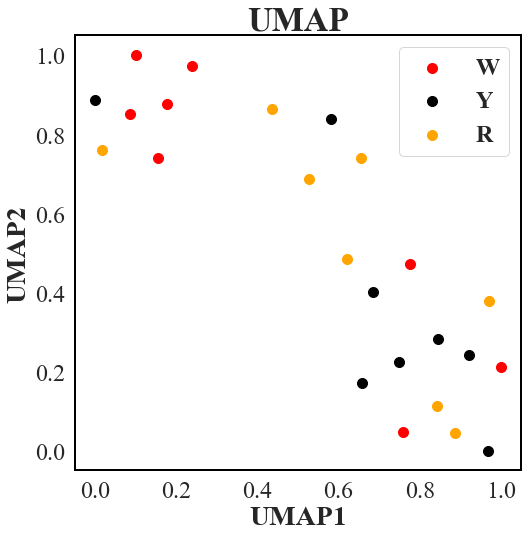

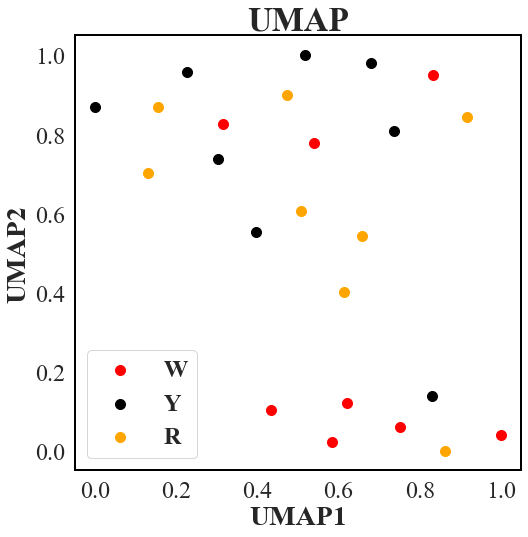

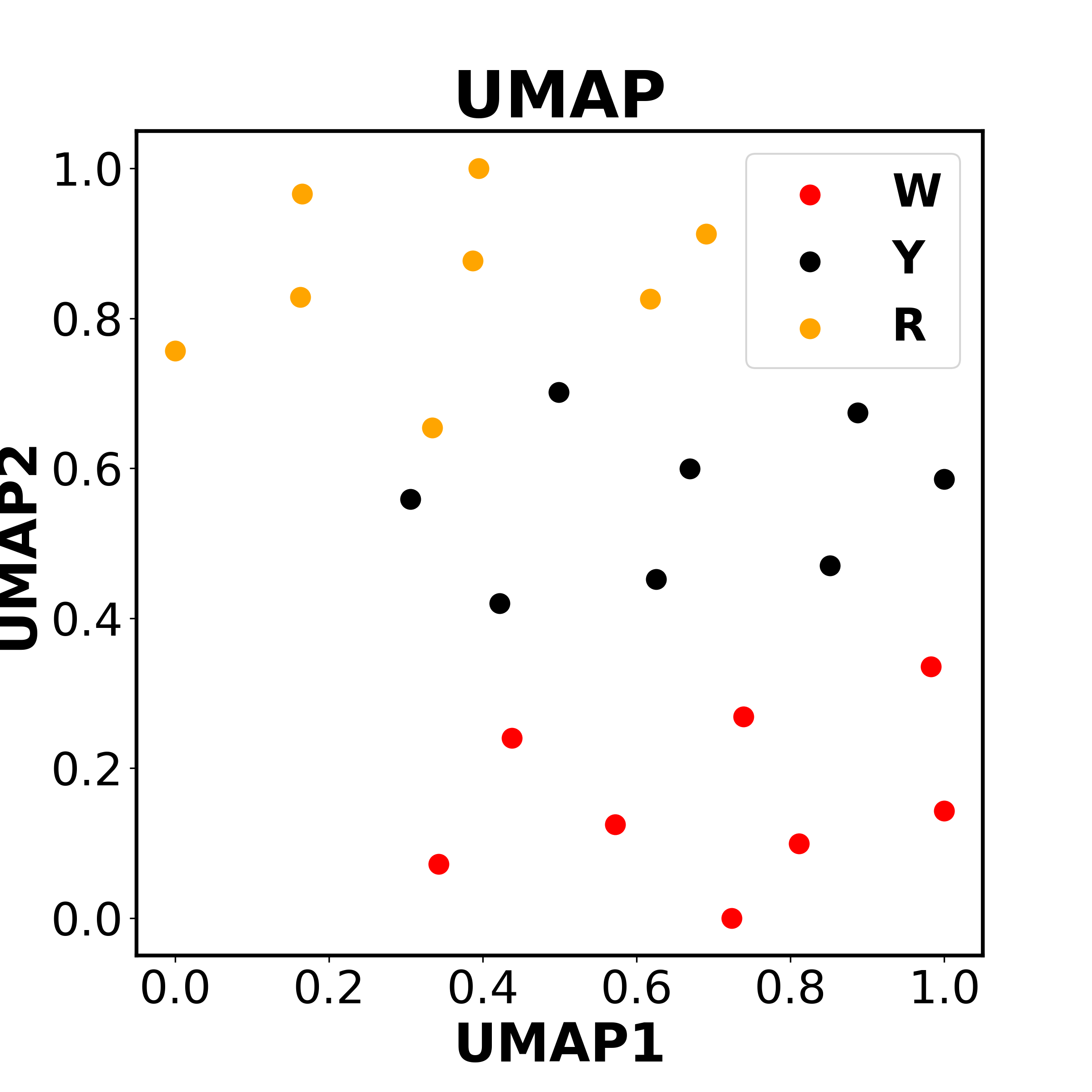

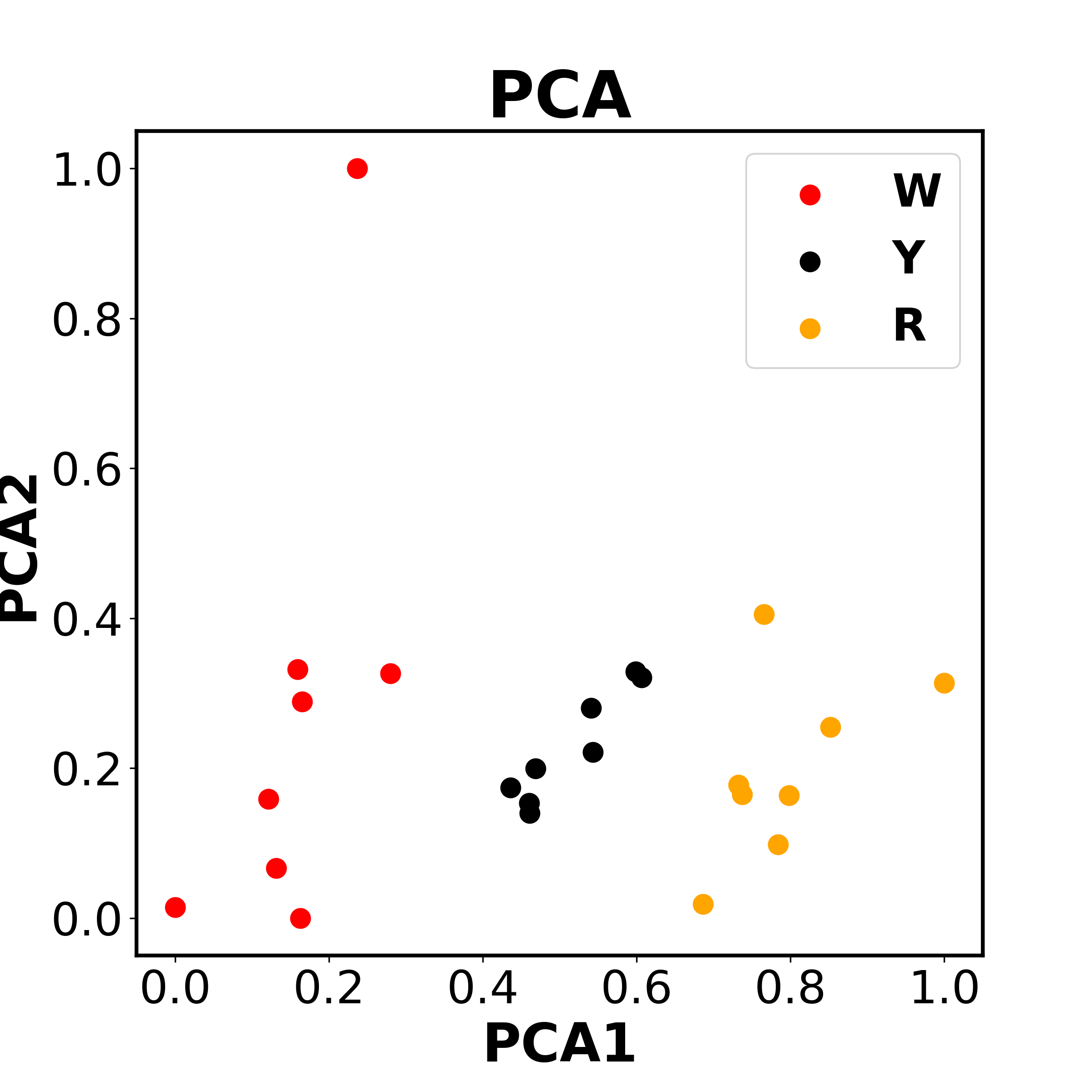

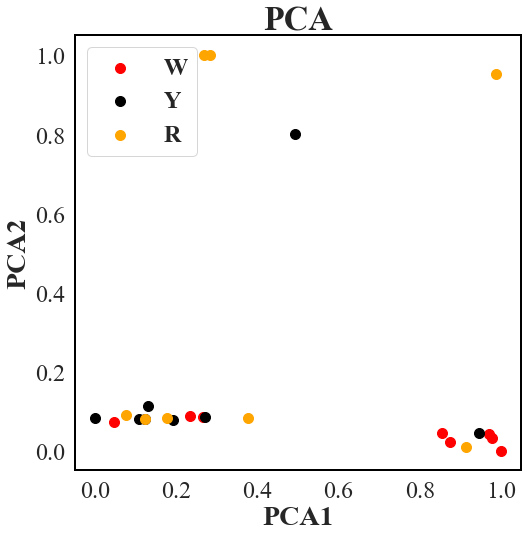

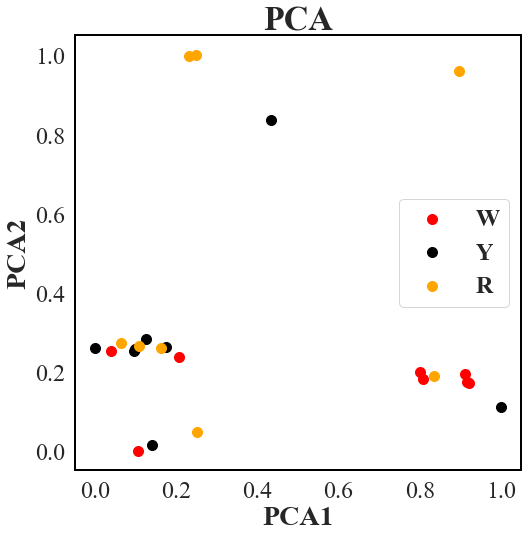


**Fig. S5** PCA and UMAP plots of 390 metabolites (A,D), 33 (B,E) and 38 (C,F) metabolites selected by the Lasso method.

**F**

**E**

**D**

**C**

**B**

**A**


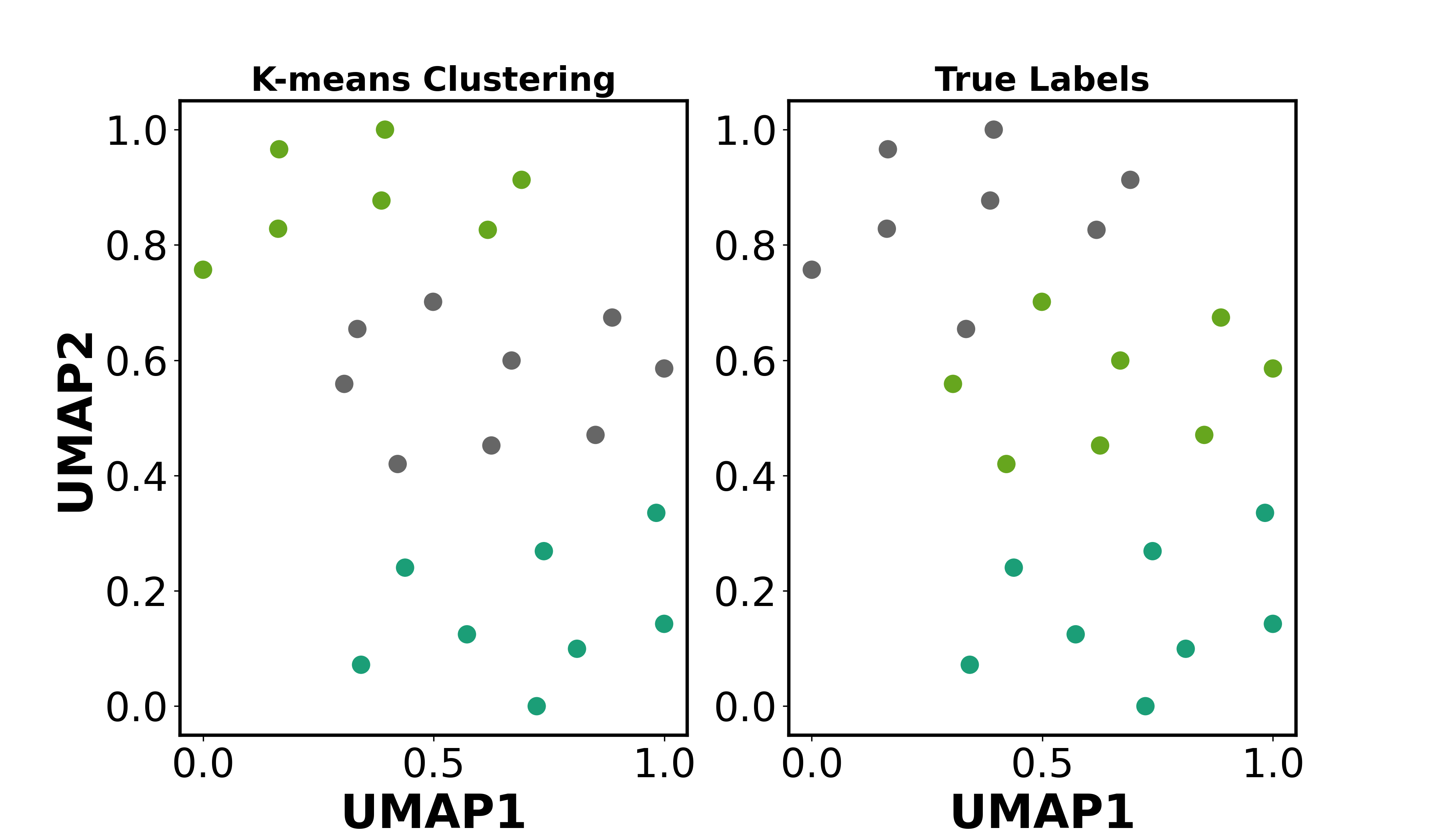

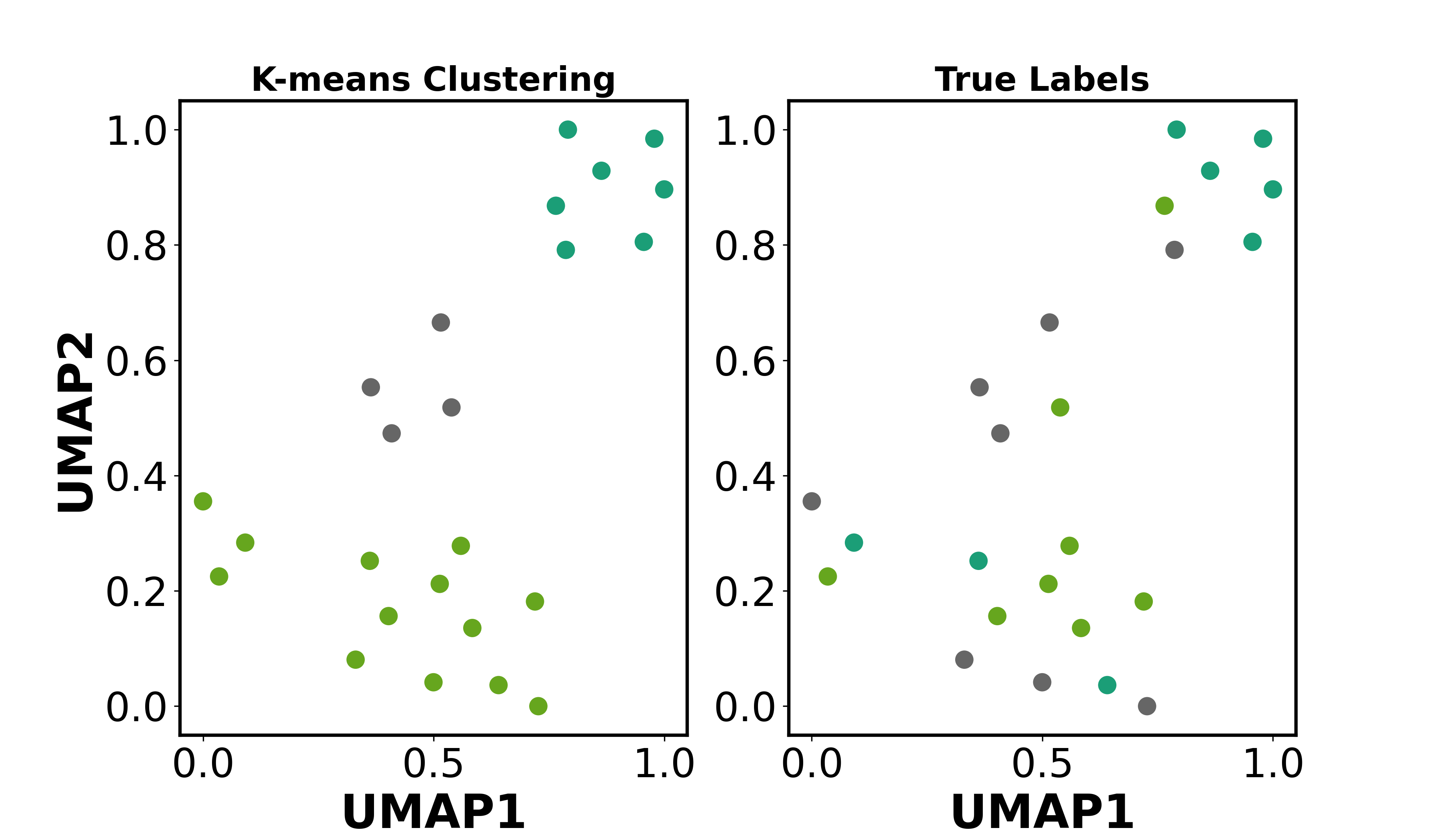


**A**

**B**

**Fig. S6 The unsupervised learning K-means algorithm was used to cluster the original data (A) of three onion species and the features (B) selected by LASSO**


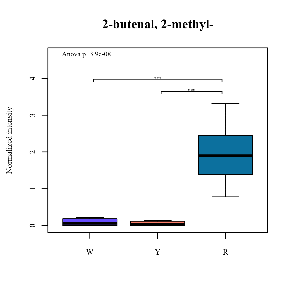

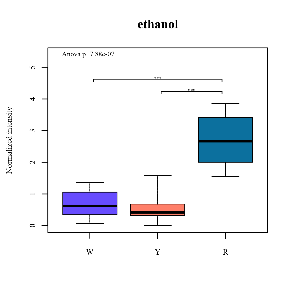

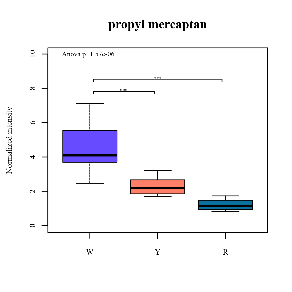

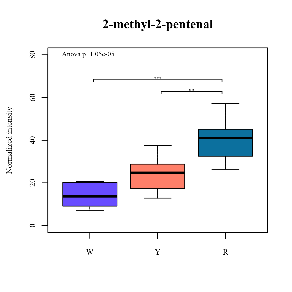

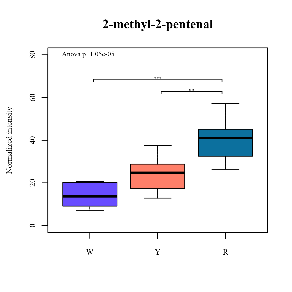

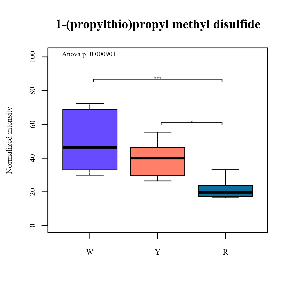

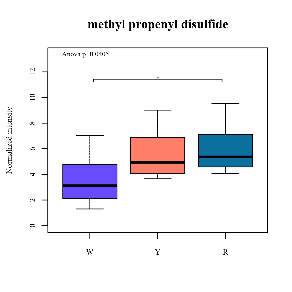

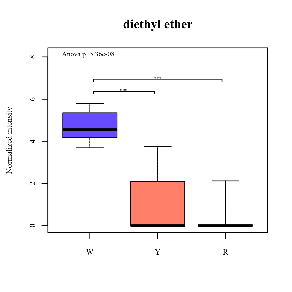

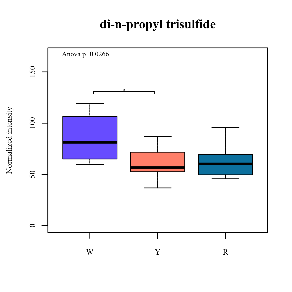
























**Fig. S7 Twenty key metabolites screened based on random forest and SHAP values. * denotes significant difference at 0.05 level. ** denotes significant difference at 0.01 level. *** denotes significant difference at 0.001 level**
